# An operational urban air quality model ENFUSER, based on dispersion modelling and data assimilation

**DOI:** 10.1016/j.envsoft.2022.105460

**Published:** 2022-10

**Authors:** Lasse Johansson, Ari Karppinen, Mona Kurppa, Anu Kousa, Jarkko V. Niemi, Jaakko Kukkonen

**Affiliations:** a Atmospheric Composition Research, Finnish Meteorological Institute, Helsinki, Finland; b Helsinki Region Environmental Services Authority HSY, Ilmalantori 1, FI-00240, Helsinki, Finland; c Centre for Atmospheric and Climate Physics Research, And Centre for Climate Change Research, University of Hertfordshire, College Lane, Hatfield, AL10 9AB, UK

**Keywords:** Air quality, Dispersion modelling, Data assimilation

## Abstract

An operational urban air quality modelling system ENFUSER is presented with an evaluation against measured data. ENFUSER combines several dispersion modelling approaches, uses data assimilation, and continuously extracts information from online, global open-access sources. The modelling area is described with a combination of geographic datasets. These GIS datasets are globally available with open access, and therefore the model can be applied worldwide. Urban scale dispersion is addressed with a combination of Gaussian puff and Gaussian plume modelling, and long-range transport of pollutants is accounted for via a separate regional model. The presented data assimilation method, which supports the use of AQ sensors and incorporates a longer-term learning mechanism, adjusts emission factors and the regional background values on an hourly basis. The model can be used with reasonable accuracy also in urban areas, for which detailed emissions inventories would not be available, due to the data assimilation capabilities.

## Highlights

• ENFUSER provides detailed and timely information on urban air quality.
• The performance of the model was evaluated in Helsinki region.
• Data assimilation is used to assist operational urban air quality modelling.
• The modelling system can be used worldwide.

## Graphical abstract

**Figure:**
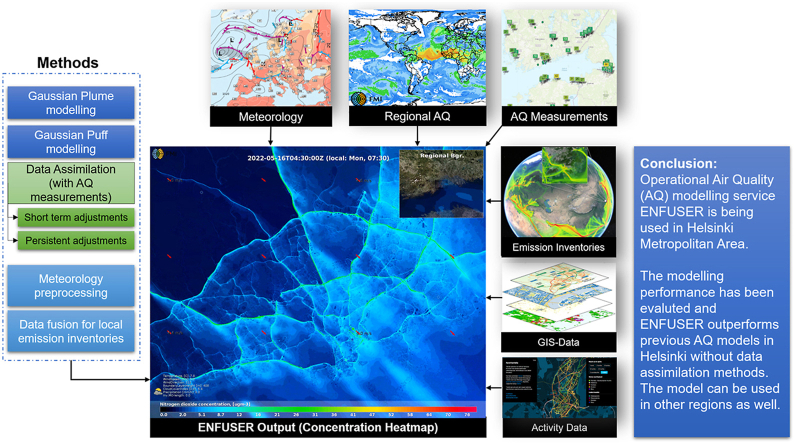

## 1 Introduction

Air pollution is a major environmental concern in many areas worldwide, having a strong impact on public health and the economy (World Health Organization, 2016; Guerreiro et al., 2014). The spatial variability of air quality is especially pronounced in urban environments. It is common for, e.g., European cities to report exceedances of annual air quality limit values at urban monitoring stations (EEA, 2018). Therefore, high-quality up-to-date information on local air quality is needed, to make it possible for citizens to assess their personal exposure to air pollutants and make informed decisions on, e.g., their commuting options. Further, local authorities also require such timely information to make decisions on effective interventions and countermeasures.

The amount and variety of data available to facilitate high-quality, local-scale air quality (AQ) modelling have been increasing steadily over the past decade, due to technological advancement and through open-access initiatives such as INSPIRE (EU, 2007). For instance, Sentinel-2 from the Copernicus programme (Gascon et al., 2014) provides open access satellite imaging, and Sentinel-5 (Veefkind et al., 2012) provides information on the atmospheric composition of air pollutants globally. The voluntary participation of citizens may also result in the accumulation of useful information. One prominent example of this trend is the use of OpenStreetMap as a source for detailed land-use information (Estima and Painho, 2013; Yang, D., Smith, A.C., and Yu, Q., 2017; Arsanjani et al., 2015). Detailed traffic emissions can nowadays be estimated by deriving road-specific driving cycles using GPS data from vehicles, and by combining this information with vehicular emission factors (Borrego et al., 2016). In harbour areas the impact of shipping can be modelled in near real-time using online AIS data (e.g., Huang et al., 2020). Moreover, new information can be derived when these datasets are processed further or combined intelligently with data fusion. As an example, Misra et al. (2020) used Sentinel-2 satellite data successfully with object detection methods to assess brick kilns around Delhi, India.

The amount and availability of AQ measurement data have also substantially increased recently. This is partly due to the introduction of online services that gather and readily provide measurement data. An example of these services is the Air Quality in China service (AQICN, 2022) for global online measurements; this service has been used, e.g., for the mapping of pollutant concentrations in China (Rohde, and Muller, 2015). Another reason for the improved availability of measurement data is the wider use of low-cost air quality sensors, which can be deployed in large quantities to complement the reference quality network of stations (Kuula et al., 2019; Petäjä et al., 2021). However, the quality of sensor measurements can change over time and be affected, e.g., by meteorological conditions (Petäjä et al., 2021). Clearly, assimilation of sensor data in air quality modelling must consider this quality issue.

The most common approach in urban scale modelling, including downscaling systems, is to utilize multisource Gaussian modelling. However, these models cannot explicitly resolve the influence of urban buildings and other obstacles on the dispersion of pollution. Various street canyon dispersion models, such as the Operational Street Pollution Model (OSPM) (Berkowicz, 2000) address this issue by approximating the circular motions of air moving within the street canyons. The performance of the OSPM has been widely evaluated against experimental data, e.g., in various cities in Denmark (e.g., Jensen et al., 2017) and in Helsinki (e.g., Kukkonen et al., 2001a, Kukkonen et al., 2001b).

Denby et al. (2020) presented a downscaling procedure, starting from the output of a regional chemical transport model (CTM), and downscaling this into a local scale (50 m) using Gaussian modelling principles. Another example of a downscaling approach has been presented for Barcelona by Benavides et al. (2019). Their approach was based on a coupling of a regional scale model with an urban scale one, CALIOPE-Urban. CALIOPE-Urban, like many other models, avoids double-counting emissions from the regional scale model, while resolving a higher resolution concentration field upwind of the grid cells provided by the regional model.

Computational fluid dynamics (CFD) models are the most accurate methods for simulating the complex wind field and dispersion of air pollutants in urban areas, but these methods are also the most resource-consuming ones. As an example, a Large Eddy Simulation (LES) model (e.g., Hellsten et al., 2020) can provide a sub-meter grid resolution, allow for the effects of buildings and canopy, and such models can be used in nested domains. The LES models cannot yet be operationally used for a full city-scale using the currently available computer technology. However, there are downscaling applications using LES methods for a limited urban area and duration (e.g., Nuterman et al., 2021). In addition to Gaussian- and CFD models, there are several different modelling approaches used in urban dispersion modelling, including Eulerian (e.g., Karl et al., 2019) and Lagrangian methods (Rotach, 2001). Karl et al. (2019) have used a combination of different types of dispersion modelling techniques and a more advanced treatment for photochemistry.

There are several urban air quality models that utilize data assimilation or data fusion of air quality measurements. Schneider et al. (2017) presented a method for the generation of high-resolution urban AQ mapping, by including dispersion modelling coupled with AQ-measurement driven geostatistical data assimilation. The basis of the method is the universal kriging technique (Goovaerts, 1997) using a so-called base map, e.g., a longer-term average concentration field. The limitations of this method include the need for a substantial amount of measurement locations, and an accurate base concentration field. Similarly, Gressent et al. (2020) used sensor data in data fusion to enhance urban air quality mapping for PM_10_ in Nantes, France. The adopted data fusion method was kriging; it requires a base map in the form of modelled annual average PM_10_ concentrations. The used sensor data is a large collection of fixed and mobile measurements, and the model therefore applies pre-processing and filtering methods.

The aim of this paper is to present an operational multi-scale air quality modelling system called ENFUSER (Environmental information fusion service). The modelling system combines several datasets regarding online information on geography, meteorology, regional background concentrations, AQ measurements and local activity datasets. The model provides adaptive, hourly high-resolution air quality modelling output for the general public, decision-makers, and other end-users. The novelty of the presented system is the combination of multi-scale dispersion modelling in urban scale operative service, and data assimilation that is not based on kriging. The adopted data assimilation method - which also supports AQ sensor data to be used - facilitates physically meaningful (i.e., a detected change in emission patterns), persistent learning mechanisms to guide the modelling. This kind of an operative model has not been presented previously.

The first objective of this paper is to present the mathematical model within the operational modelling system, including the approaches to combining Gaussian plume- and puff modelling techniques with data assimilation. The second objective is to present the technical solution to perform the high-resolution computations at a moderate computational cost. The third objective is to present a model evaluation in the Helsinki Metropolitan Area (HMA) in Finland for two selected years. The fourth objective is to present a non-kriging-based data assimilation approach for urban-scale air quality modelling.

## 2 Materials and methods

### 2.1 Overview of the modelling system

ENFUSER is an operational local scale air quality model (a combination of Gaussian puff & plume) used in the Helsinki Metropolitan area in Finland (HMA). The model has also been used in foreign installation sites such as Nanjing, China and Delhi, India. The current set of modelled pollutant species in HMA includes nitrogen dioxide (NO_2_), ozone (O_3_), fine particles (PM_2.5_) and thoracic particles (PM_10_) for which the model provides hourly average concentrations at a breathing height of 2m above ground. The model also produces derivative output such as the Finnish national Air Quality Index (AQI) based on the primary modelling output (i.e., the pollutant concentrations).

**Fig. 1:**
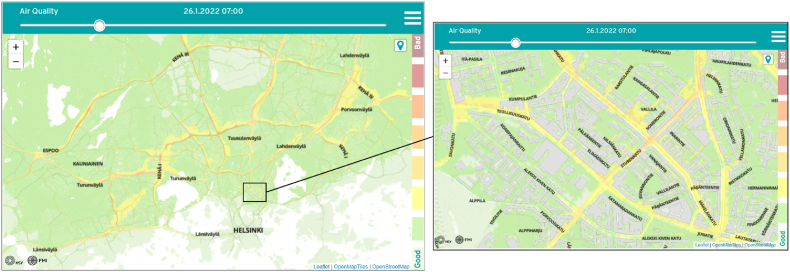
Example of air quality index visualizations provided for the general public based on ENFUSER model data. The service can be accessed from: https://www.hsy.fi/en/air-quality-and-climate/air-quality-now/air-quality-map/. Visited: Jan 26th, 2022.

The output provided by the model is publicly available via FMI's Open Data portal (FMI, 2021). Hourly updating AQI visualizations based on the model results are available at (HSY, 2022) but also shown in public transportation displays in HMA; a caption of the service is shown in Fig. 1. Further, there is also a separate service providing annual average information based on historical model data (HSY, 2021). Modelling results are updated each hour, each time including a now-casting period with measurements 12h in the past and a forecasting period to the future (12h) in a resolution of 13 × 13m, covering an area of approx. 40 × 30 km.

Historically, ENFUSER is a predecessor of the land use regression-based model presented in (Johansson et al., 2015). With respect to this previous work, the aim of incorporating air quality measurements for improved urban air quality predictions has remained the same, however, the land-use regression-based approach has been replaced with a more realistic dispersion modelling approach.

**Fig. 2:**
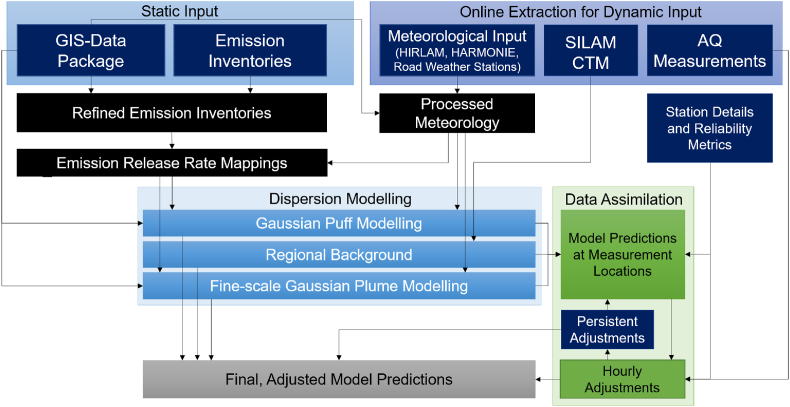
Schematic diagram for the ENFUSER model. Dark blue elements describe the input datasets (static and continuously extracted dynamic online data). Black elements stand for intermediary data sets that are processed from input and facilitate dispersion modelling. Light blue elements show the three layers of dispersion modelling that are computed separately. Green elements are intermediary datasets and outcomes of measurement-based data assimilation. The final modelling outcome, which is a gridded pollutant concentration field, is data as an assimilation-adjusted combination of the three layers of dispersion modelling.

A schematic presentation of the modelling system has been shown in Fig. 2. More thorough descriptions of the individual components presented in the figure are given in the following sections of the paper. In brief, the input datasets, which can be separated into static and dynamic ones, are shown at the top of the diagram. The static input data is used for the description of the modelling area and local emission inventories. Often the available emission inventory data is not directly useful for local-scale modelling, and therefore the modelling system may need to refine or downscale emission inventories based on GIS data. The dynamic input consists of meteorological data (both numerical and measured), regional AQ forecasts and AQ measurements; this set of input is continuously extracted from online sources. The raw meteorological input is not sufficient for local-scale dispersion modelling and pre-processing of meteorology is therefore used.

The dispersion modelling operates in three different layers: Gaussian puff (urban-scale), regional background (regional-scale) and local-scale Gaussian plume modelling. For the Gaussian puff and plume modelling the underlying emission information is the same, and the model must index and represent the emission release data in different resolutions simultaneously. The meteorological conditions may also affect hourly release rates of emissions (e.g., ambient temperature and emissions from residential wood combustion). The dispersion modelling in the three separate layers is combined to form model predictions at measurement locations. These predictions will then be used in data assimilation (a procedure described in Section 2.4) which results in a set of hourly adjustments that will help the model to obtain a higher degree of agreement at the measurement locations. This process requires a light database for measurement location specifics, including device quality ratings, exact coordinates, and the measurement height.

Once data assimilation-based adjustments have been assessed for a given hour and a given pollutant species, the final model predictions can be computed for the overall modelling area. Each data assimilation outcome that occurs during the now-casting period (i.e., the period, for which the air quality measurement data is already available) will update a more gradual, persistent set of adjustment factors (PA). The PA factors are considered also during the forecasting period that extends up to 12h to the future; clearly, no measured air quality data is available for this period at the time of computing the forecast. When a new modelling task launches the previous state of PA is loaded as input; when a modelling task is completed the updated PA is stored locally to be used for subsequent modelling tasks.

### 2.2 Input data for the model

**Fig. 3:**
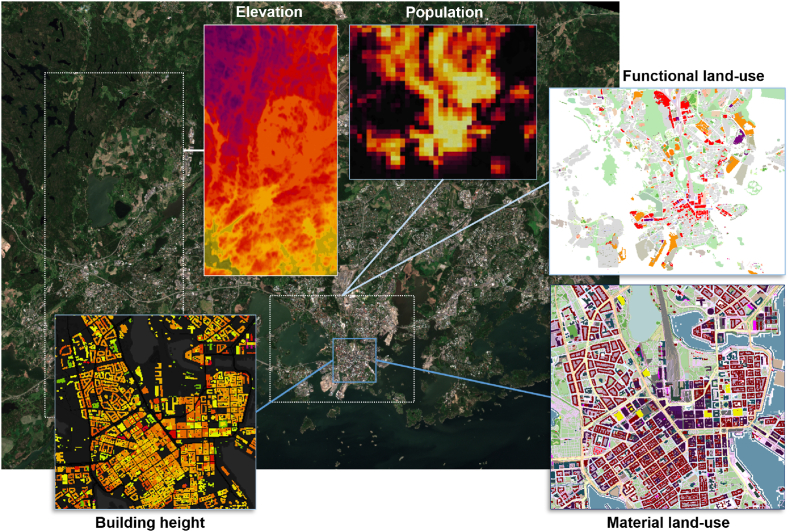
Illustration of different GIS datasets used by ENFUSER in the Helsinki modelling region. Background satellite image: Copernicus Sentinel 2-MSI (2020, 10m). Functional (10m)- and material (5m) land use based on OSM data. Population data (300m) obtained from Global Human Settlement, Elevation from NASA SRTM 30m. Building height (5m) based on OSM data. The given values, e.g., 30m, stands for spatial resolution in meters for the datasets.

The input datasets required by the model can be separated into static and dynamic input datasets. To set up new modelling domains (e.g., a collection of cities in a selected country), certain GIS datasets need to be extracted from various online sources. These extracted online GIS datasets (Fig. 3) are updated periodically (e.g., annually), however, due to the low frequency of updates these GIS datasets are referred to as ‘static’ input. For this GIS-data extraction and the preparation of static datasets, there is an automatic procedure built-in ENFUSER.

A dedicated sub-program is used to access various online sources for dynamic input. This sub-program continuously extracts and archives recent data locally for all modelling areas that have been defined in ENFUSER. The sub-program is always kept active and provides the model with up-to-date input data without temporal gaps. When the model is used for AQ predictions, this local archive of dynamic data is accessed. In HMA the dynamic information includes the following: reference quality AQ data from 12 measurement stations (FMI, 2021), regional-scale AQ forecasts from the chemical transport model SILAM (Sofiev et al., 2015), HARMONIE Numerical Weather Prediction (NWP) data with 1 × 1 km resolution for meteorology (Bengtsson et al., 2017) and road weather measurements (DigiTraffic, 2021). Additionally, traffic flow measurements (DigiTraffic, 2021) and traffic congestion data are being extracted continuously. However, these additional information sources are used in supporting offline tasks^1^ and do not yet contribute to the operational modelling outcome.

#### 2.2.1 Geographical information system (GIS) data

ENFUSER has a built-in capability of extracting and processing global, open-access geographic information for its modelling areas. There are several use-cases for this information, e.g., during the dispersion modelling information on ground elevation, urban structures and surface roughness length estimates are needed. GIS-based derivatives are often needed as global coarse-resolution inventories such as EDGARv5 (Crippa et al., 2019) cannot be used without post-processing and downscaling in local-scale modelling.

The OpenStreetMap raw data (OSM, 2021) can be considered as the foundation for the GIS datasets that are used to describe the modelling areas. This provides the model with a detailed high-resolution land-use mapping that is especially useful for the description of road networks and the layout of buildings. The modelling area is described with OSM data in two layers: the functional land-use (e.g., ‘parking space’) and the material land-use (e.g., ‘asphalt’). For buildings and roads, the model uses an object-oriented mapping that can e.g., describe the road name, number of lanes, speed limits, etc. Another source of information is the Global Human Settlement (EU, 2021), which provides additional land-use- and population data.

The ground elevation is extracted from NASA SRTM and contributes to surface roughness estimations and height parameters used in dispersion modelling. 10 × 10m multi-spectral satellite imagery is extracted from Copernicus Sentinel-2 MSI. These above-mentioned sources can be regarded as the primary sources for GIS data for the model and based on them additional derivative datasets are proxied. One such derivative is shown in Fig. 3, the building height dataset. The building height dataset is estimated based on the OSM building characteristics, which often specify the height of the building in meters, or the number of floors. However, for many buildings, this characterization does not exist, and therefore the height can only be roughly estimated based on the building type and building surface area.

#### 2.2.2 Emission data

It has previously been found that the most important urban source categories in HMA are residential wood combustion and vehicular traffic (Kukkonen et al., 2018 and 2020). For shipping and harbour activities, the contribution to the total concentrations of PM_2.5_ in the three-year period 2012–2014 has been estimated to exceed 10% only in the vicinity of major harbours (Kukkonen et al., 2018). The contribution of power production and industrial activities to local PM_2.5_ concentrations has been negligible in most parts of the area. Leisure boat emissions for the coastal area of HMA can be estimated with the BEAM model (Johansson et al., 2020) but these presumably negligible emission contributions have not been included.

**Fig. 4:**
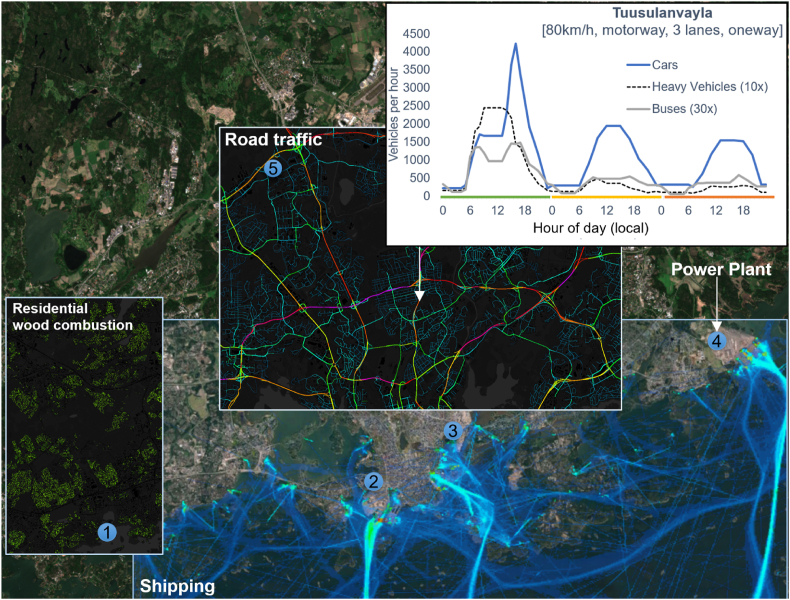
Illustration of the most notable emission inventories and traffic flow mappings in Helsinki. All the emission source categories cover the whole modelling area and only parts of them have been shown for visual clarity. In the top right corner, the daily average total car count for roads has been shown (cyan: 400, purple: 70 000 cars/d). At the centre, the estimated average hourly traffic flow profile for a selected road element is shown (green = working days, yellow = Saturday, orange = Sunday). For visual clarity, the hourly flow values for heavy vehicles (10x) and Buses (30x) have been scaled higher. Power plant locations have been shown with a light blue dot. Background satellite image: Copernicus Sentinel 2-MSI (2020). Illustrative shipping emissions are shown based on STEAM3.

In ENFUSER the basis for most emission inventories is a gridded annual- or monthly emission total dataset, for which a temporal profile to sequence emission release rates has been defined. In addition, there are a), road traffic emissions (based on OSM-road network structure coupled with vehicle flow counts) and b), shipping emissions (high-resolution dynamic emission inventory given by the STEAM3 model (Johansson et al., 2017) and c), power plants and factories (treated as individual entities that are modelled as elevated point sources). The most notable emission sources for HMA have been illustrated in Fig. 4.

##### Road traffic emissions

The road traffic emission modelling is based on the OpenStreetMap road network. Each piece of a road (of which there are tens of thousands in HMA's dataset) is treated as a separate entity with characteristics such as speed limit, road type, number of lanes and surface material. Hourly vehicle flow patterns are attached to these road elements and then an emission release rate as [µg/m^2^/s] is computed based on the vehicle type, road speed limit and the number of vehicles flowing through the road at the time of assessment. There are three classes for vehicle types: cars, buses and heavy vehicles. To define these emission factors, flow-speed relationships from (Kristensson et al., 2004) and vehicle category-specific unit emission factors from (VTT, 2021) have been used. An averaged vehicle flow pattern with 72 hourly values (24 for Monday to Friday, 24 for Saturday, 24 for Sunday, as shown in Fig. 4) is implemented for each road segment, separately for each vehicle class. In addition, a weekly correction factor can be applied to address e.g., holiday seasons.

A dataset given by local authorities in Helsinki (Helsinki Region Transport HSL and the City of Helsinki, using the EMME^2^ model) has been used for defining these averaged traffic flow patterns. This material describes averaged hourly flow counts as line data (>30 000 lines). The vehicle flow information characterized by the line data has then been implemented to the road network described by the OSM data. For each OSM road segment, a nearby EMME line element is searched with matching orientation and speed limits. For lesser roads for which a corresponding EMME characterization is not found, a statistical approximation is used instead, based on GIS data and a generic temporal profile obtained from nearby roads. This procedure of implementing vehicle flow patterns from line input data into the OSM road network is a supporting feature of ENFUSER and can be used also in other areas, in addition to Helsinki.

##### Road dust emissions

Complex deterministic models have previously been developed for the evaluation of suspended dust from the road and street surfaces (e.g., Kauhaniemi et al., 2011; Denby et al., 2013; Kauhaniemi et al., 2014; Stojiljkovic et al., 2019). However, such models require a comprehensive set of input data, for instance, data on the road sanding and the use of studded tires. Such data is commonly not operationally available. We have therefore applied a simpler statistical method for the evaluation of suspended dust.

The modelling of road dust and resuspension of particles is based on the data on the road network, vehicle flow speed and hourly flow counts. Also, the effects of road surface moisture, the usage of studded winter tires and other meteorological factors are considered to assess the resuspension of coarse particles (defined as PM_10_ - PM_2.5_). Road surface moisture estimates are obtained via (DigiTraffic, 2021), and a generic weekly profile for the usage of studded tires in HMA is used.

##### Power plant emissions

Power plants are treated as a collection of elevated point sources, each being modelled solely with Gaussian puff dispersion. In ENFUSER generalized profiles have been created for coal, oil and natural gas power plants that specify emission factors in terms of [g/MWs] with monthly/diurnal variability. The set of power plants nearby the modelling area is based on (the Global Energy Observatory, 2021) which specifies the location, type, and capacity (MW) of the power plants. In the HMA there are 5 combined heat and power plants that are shown in Fig. 4. More specifically: 1), Suomenoja (coal and natural gas) 360 MW, 2), Salmisaari (coal and wood pellets) 160 MW, 3), Hanasaari-B (coal and biomass) 220 MW, 4), Vuosaari (natural gas) 630 MW and 5), Martinlaakso (biomass and natural gas) 197 MW.

##### Shipping emissions

Shipping emission inventories for Helsinki are provided by the STEAM3 model (Johansson et al., 2017) using historic Helcom AIS data by the courtesy of the Riparian states of the Baltic Sea and a vessel database from IHS-Fairplay. The shipping emission inventory given by STEAM3 describes ships, their positions, time stamps, emission release rates [g/s] and emission release heights. The detail at which shipping emissions are represented and delivered to ENFUSER facilitates the modelling of shipping emissions as moving point sources while considering the effective emission height. However, in operational use a less ambitious resolution of approx. 100 × 100m in 5-min temporal resolution is used to represent shipping emissions.

##### Residential wood combustion emissions

An unprecedentedly detailed emission inventory for residential wood combustion (RWC) has been compiled in the HMA. Kukkonen et al. (2020) used this emission inventory to model deterministically the PM_2.5_ concentrations, including RWC. This study also evaluated the concentrations of PM_2.5_ and the related contributions of RWC in three other Nordic cities, viz. Copenhagen, Oslo and Umeå. For a more detailed description of this inventory and its use in dispersion modelling, the reader is referred to Kukkonen et al. (2020).

The above-mentioned emission inventory for RWC was also used in the present study. The inventory has three components, viz. heating, saunas, and fireplaces, each of which has different diurnal patterns and different dependencies on the ambient temperature. Clearly, residential heating activities are increased during low ambient temperatures in winter, especially for the heating component; when the ambient temperature is lower than 14 °C, the modelling assumes that the emission factor for residential wood combustion is increased in proportion to the difference of the actual temperature and the reference value of 14 °C. The modelled temperature dependencies have been derived based on the monthly total emissions from RWC and the monthly average temperatures. The emissions are presented with a resolution of 20 × 20m (Fig. 4) and the release height of the RWC emissions has been assumed to be 10m.

### 2.3 Atmospheric dispersion modelling

The model applies a multi-scale approach: a) local-scale assessment using Gaussian plume modelling, b) urban-scale assessment using Gaussian puff modelling and c) regional scale background extracted from the outputs of a chemical transport model. The contribution of each of these three is assessed and combined at a selected receptor point (RP). To avoid double-counting of emissions while combining Gaussian plume and puff modelling, the distances of emission sources from RP and the travel distances of Gaussian puffs are considered.

The pollutant concentration C(x,t) for a selected pollutant species prior data assimilation at location **x** and time t (at the receptor point, RP) is given by:

(1) $$C\left(x,t\right)=\sum_{n\;\in \;N}\sum_{k\;\in \;K}c_{L}\left(e_{k}\left(t,x_{n}\right),x\right)\;+\sum_{p\;\in \;P}c_{P}\left(p\left(t\right),x\right)+c_{BG}\left(x,t\right)$$

where N is a set of incremental locations around the RP, covering the surface area up to a selected maximum distance of $d_{cut\;}$. K is the set of incremental emission sources at the location n, for which the emission release rate in terms of [µg/s] for the selected pollutant species is $e_{k}\left(t,x_{n}\right)$. $e_{k}$ also characterises an emission release height [m] and an emission source category it is associated with (e.g., traffic, RWC, etc.). The concentration at RP caused by $e_{k}$ in terms of [µg/m^3^] is given by $c_{L}$. $c_{P}$ refers to the concentration evaluated using the Gaussian puff approach at RP, for which there are a total of P puffs to address. During the evaluation time t, each puff has a specific state p(t) that stands for, e.g., the centre point location and the carried pollutant mass, [µg], which are needed for the assessment of $c_{P}$. The final term, $c_{BG},$ is the regional background concentration, excluding the influence of the local and urban emissions.

The size of the set N depends on the resolution, in which the local emission information around the RP is represented, and from the maximum assessment distance $d_{cut\;}$. In the model, the finest used resolution to represent incremental emission sources around RP is 5 × 5 m and the size of N is of the order of several thousand. For each of these incremental areas n, there can be multiple emission sources (K) but this is often limited to only a few such emission sources; most often the set K is empty (i.e., no emissions).

The parameter $d_{cut\;}$ is the cut-off distance, at which the modelling approach switches from Gaussian plume into Gaussian puff modelling. Therefore, the set P includes only the puffs that have travelled at least the distance of $d_{cut\;}$. A low value of $d_{cut\;}$ makes the modelling more agile (i.e., can more realistically address rapidly changing meteorological conditions) but requires that the puff modelling is performed in higher detail (this will result in an increase of the computational cost). Considering the technical hardware limitations of the modelling configuration and based on a collection of simulations with different values for $d_{cut\;}$, a value of 2000m has been selected to be used in the HMA.

#### 2.3.1 Gaussian plume computations

Following the work in (Seinfeld and Pandis, 2016) the Gaussian plume solution with steady-state meteorology, totally reflecting ground is given by

(2) $$c_{L}=\frac{Q}{2\pi u\sigma_{y}\sigma_{z}}\exp\;\left(-\frac{y^{2}}{2{\sigma_{y}}^{2}}\right)\;\left[\exp\;\left(-\frac{{\left(z-H\right)}^{2}}{2{\sigma_{z}}^{2}}\right)\;+\;\exp\;\left(-\frac{{\left(z+H\right)}^{2}}{2{\sigma_{z}}^{2}}\right)\;\right]$$

where $c_{L}$ is the concentration at RP, Q is the emission rate [µg/s], the standard deviation $\sigma_{y}$ defines the spread of the dispersion horizontally and $\sigma_{z}$ vertically. Variable u is the wind speed [m/s], y is the horizontal perpendicular distance from the line aligned with wind direction, H is the emission height [m] and z is the height of the receptor point above ground [m]. Vertical profiles for wind speed or diffusivity are not considered.

Eq. (2) does not address the influence of a finite boundary layer height or the settling velocity of particles. The model, however, includes these effects by using extended versions of Eq. (2); for readability these have been presented in Appendix A. Computationally efficient ways to implement these equations in practice have been presented in supplements.

#### 2.3.2 Estimation of Gaussian standard deviations

There are several empirical approaches for the assessment of the Gaussian standard deviations $\sigma_{z}$ and $\sigma_{z}$ (McElroy, 1968; Smith, 1973; McMullen, R.W., 1975). The approach from (Briggs, 1973, p. 124) is adopted, in which the standard deviations are estimated as a function of distance (x,[m]) from the emission source along the wind direction but depending on a stability classification. These empirical formulas predict standard deviations $\sigma_{y}\left(x\right)$ and $\sigma_{z}\left(x\right)$ defined separately for a discrete set of different atmospheric stability conditions ([A, B, C, D, E, F], ranging from highly unstable (A) to extremely stable (F)). The stability class is selected based on the meteorological conditions (Mohan and Siddiqui, 1998). Specifically, the Monin-Obukhov length -based method is adopted.

Traffic-produced turbulence (TPT) can become a dominant cause for mixing and the dilution of pollutant concentrations nearby roads, especially in low wind scenarios. The inclusion of TPT in an urban scale model can be challenging; in (Solazzo et al., 2007) several TPT schemes have been evaluated. In (Härkönen et al., 1996) a semi-empirical treatment is used by introducing additional standard deviation terms $\sigma_{y0}$ and $\sigma_{z0}$ as a function of wind speed and the angular difference between the road and the wind. However, in (Härkönen et al., 1996) the vehicular flow speed is ignored, whereas it is considered in (Solazzo et al., 2007). In ENFUSER, TPT is addressed simply by enhancing the diffusion of pollution by increasing the values of $\sigma_{y}\left(x\right)$ and $\sigma_{z}\left(x\right)$ as a function of traffic flow speed while the vehicle density is not considered. For example, the value of x for the assessment of σ(x) is increased by 10m for a motorway emission source (with a flow speed of 120 km/h). In essence this introduces additional mixing in a similar way that the addition of $\sigma_{y0}$, $\sigma_{z0}$ does. The used parametrization requires further research, however; the primary aim of this simplified approach is to remove outliers (overprediction) in the immediate vicinity of main roads and the simplistic parametrization achieves this.

#### 2.3.3 Wind profiling approach

The following method is used for the assessment of wind speed at the height of the emission source u(H): first, the model takes interpolated data from the HARMONIE NWP model at a height of 10m (u(10)). This interpolation considers both spatial and temporal dimensions, and therefore provides smoothly variating preliminary wind fields. Then u(H) is evaluated based on a log-wind profile (Holmes and Bekele, 2001):

(3) $$u\left(H\right)=u\left(10\right)\frac{ln\left[\left(H-d_{0}\right)/Z_{eff}\right]}{ln\left[\left(10-d_{0}\right)/Z_{eff}\right]},\;u\left(H\right)\geq 1$$

where $d_{0}$ is the zero-plane displacement (m) and $Z_{eff}$ is the effective upwind surface roughness length (m). For the assessment of $Z_{eff}$, an upwind directed assessment of land-use information is used, and the land-use information is used for the selection of a roughness length estimate. More information on the wind profiling approach and the estimation of surface roughness is presented in Appendix B.

#### 2.3.4 Gaussian plume computations in practice

A concentration field for the modelling area, for a given pollutant species and time, is estimated by applying Eq. (1) repeatedly by changing the location for the RP each time. The assessment of the N surrounding incremental areas around the RP - and their potential concentration contribution at RP if the set K is not empty - is critical for the computational performance of the model. To make this process efficient, a method has been developed to reduce the number of areas assessed this way.

**Fig. 5:**
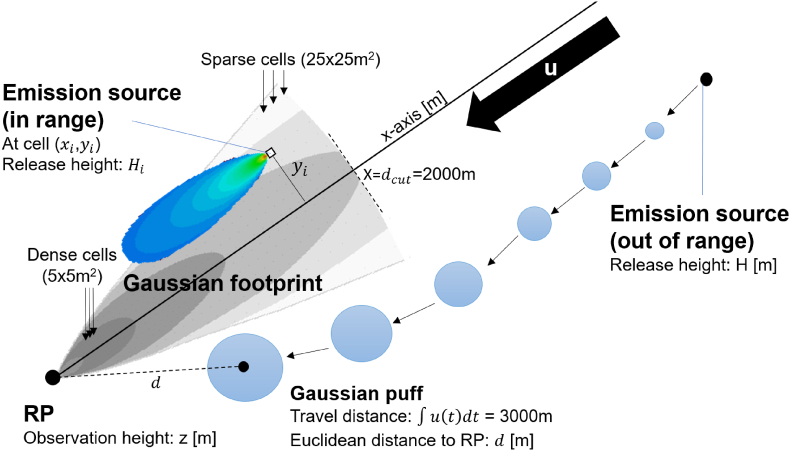
Illustration of the gaussian footprint-based approach. On the left-hand-side, the footprint is presented; it has been placed at RP and rotated to the upwind direction of the wind (u). The footprint consists of a large set of cells with varying resolutions and covers an area up to the specified maximum distance. On the right-hand-side, an emission source is shown but it does not overlap with the footprint (i.e., is farther away than $d_{cut}$), and its contribution is therefore accounted for via Gaussian puff modelling. In contrast, an example emission source at the cell ($x_{i}$, $y_{i}$) is within the footprint and its contribution at RP is assessed via Gaussian plume modelling.

A Gaussian plume-shaped footprint that consists of a large set (>1000) of incremental area cells has been shown in Fig. 5. The footprint has been designed to be rotated upwind in a single operation and define a reduced number of incremental areas for the assessment of the first term of Eq. (1). The coordinate system is like the one used with the Gaussian plume computations but reversed; the footprint is rotated to the upwind direction; the origin (x=0,y=0) is at the RP.

As shown in the figure, the incremental area cells that form the footprint have different assessment resolutions. The highest resolution is used for the cells that are close to the RP along the wind direction. To facilitate the variable assessment resolution this way, the emission information is also presented in different aggregation resolutions. For example, the cells with high resolution can access emission information at a high resolution and the cells farther away can access aggregated emission information that matches the coarser cell resolution better. Further, the footprint's cells' fixed geometry with respect to RP facilitates the use of tabled precomputed values for the assessment Eq. (2). This process, and how the footprint has been formulated, has been presented in supplements. Briefly put, each cell can instantly access the set K wherever it has been rotated on top of; for each emission source revealed this way, their concentration contribution at RP can be estimated using tabled precomputed values, excluding the term Q/u. The net contribution of all incremental emission sources is aggregated at the RP as is described in Eq. (1).

#### 2.3.5 Methods for considering the influence of buildings and street canyons

Part of the receptor points of modelling, including several measurement stations, are in complex urban environments, such as in street canyons or the vicinity of high buildings. The model surveys the nearby environment for urban obstacles (e.g., based on the locations of buildings in the GIS dataset) for each receptor point. The model subsequently archives the distances to various buildings and other obstacles, and the height of each obstacle in each direction with an estimation radius of 100m.

Based on the scanned obstacles around the RP, the model then evaluates the additional effect on dispersion caused by them. Specifically, for the computation of $c_{L}$ with the Gaussian footprint method, a limited number of area cells close to RP can be re-rotated within urban street canyons. The rotation angle in this empirical method that uses the assumptions presented in (Berkowicz, 2000), is based on the street canyon geometry and ambient wind direction. To put it simply, the re-rotation of the footprint within street canyons is to guarantee that the original surface area for local pollution in the street canyon is being accounted for regardless of the wind direction; more realistic concentration predictions can then be given for both the leeward- and windward sides of the canyon. The strongest impact of this method is observed when the wind direction is perpendicular to the street canyon. Conversely, the method has a negligible effect when the wind direction is parallel to the street canyon. As a limitation, the method cannot achieve homogeneous concentration fields within the street canyon in low wind-speed scenarios, as is assumed to occur (Berkowicz, 2000).

#### 2.3.6 Gaussian puff computations

A Gaussian puff approach is used to represent a single emission mass parcel that moves dynamically in the modelling area. The concentration caused by a Gaussian puff can be computed with (Stockie, 2011; Seinfeld and Pandis, 2016):

(4) $$c_{P}=\frac{Q_{m}}{8{\left(\frac{1}{2}\pi \sigma_{y}\sigma_{z}\right)}^{3/2\;}}\exp\;\left(-\frac{{\left(x-ut\right)}^{\;2}+y^{2}}{2{\sigma_{y}}^{2}}\right)\;\left[\exp\;\left(-\frac{{\left(z-H\right)}^{2}}{2{\sigma_{z}}^{2}}\right)\;+\;\exp\;\left(-\frac{{\left(z+H\right)}^{2}}{2{\sigma_{z}}^{2}}\right)\;\right]$$

where $Q_{m}$ is the emission mass [µg] of a pollutant species within the puff and t is the travel time. However, Eq. (4) does not support the non-linear movement, determined according to the location- and time-dependent wind field. Therefore, the following refinements that extend the applicability of Eq. (4) are adopted: first, the travel distance [m] of each puff is given by ∫u(t)dt and this is used for the computation of standard deviations $\sigma_{y},\;\sigma_{z}$. Second, a substitution ${\left(x-ut\right)}^{2\;}+y^{2\;}=d^{2\;}$ is used for Eq. (4). The term $d^{2\;}$ is the squared Euclidean distance from RP to the current centre point location of the puff (Fig. 5). After these refinements the concentration contribution assessment of a Gaussian puff at RP allows the meteorological conditions imposed for the puff to be updated on a minute basis (Appendix B), but the travel distance and centre-point need to be dynamically tracked. Further, the variables x and y have been eliminated from the equation, and the substituting variable d can be assessed in a fixed coordinate system instead of a rotated one.

The tracking of the puff travel distance has another important use case: the selection of puffs to be considered in Eq. (1) (set P) are determined based on their travel distances to avoid double counting of emissions. The set P can therefore be formed by selecting the puffs for which the travel distance exceeds $d_{cut}$. As with Gaussian plumes, extended equations are applied with Gaussian puff modelling, which considers the influence of a finite mixing layer height and the gravitational settling velocity of coarse particles (Appendix A).

The management of emission source information for the Gaussian puff modelling is different than was presented for Gaussian Plume. First, the high-resolution emission source representation is aggregated to much larger 500 × 500m sized cells, and these cells are used to frequently release emissions into the atmosphere in the form of puffs. For each puff emitted this way the source category (or several categories) information is maintained. Secondly, selected emission sources such as power plants are treated as point emission sources and dispersion modelling is solely based on Gaussian puff; no spatial aggregation is applied for such point sources, and these are not considered with Gaussian footprint-based approach.

The efficiency of assessment of Eq. (4) is important for the model's performance, and the set of puffs that are being assessed for each RP needs to be kept as low as possible. Usually, the number of puffs (set P) being modelled in HMA at any given time is in between 100 000 to 300 000. In supplements several techniques for reducing the computational burden for Gaussian puff modelling in ENFUSER have been presented.

#### 2.3.7 Regional background

The regional background is included by using hourly predictions from the operational chemical transport model SILAM (Sofiev et al., 2015). Clearly, a double-counting of emissions must be avoided; previously various methods for that have been presented, e.g., by Denby et al. (2020) and Benavides et al. (2019). The combined use of chemical transport models and urban scale dispersion models for a wide range of models has also been discussed by Kukkonen et al. (2012).

The adopted technique for assessing the regional background concentration is comparable to an upwind assessment of the background but utilizes the Gaussian puff modelling technique. The SILAM predictions are assessed at the modelling area boundaries, in which numerical background concentration markers are being continuously released and their movement tracked afterwards. These markers - that contain the regional background concentration values for all modelled species at the time of release - will travel to, from and within the modelling area according to the wind fields. For assessing the regional background for an RP at any given time, a (inverse-distance weighted) kriging interpolated value of the markers found nearby the RP are used.

#### 2.3.8 Chemistry correction to model predictions

Methods have been presented in the literature for including chemical reactions to Gaussian models. For instance, Kukkonen et al., 2001a, Kukkonen et al., 2001b and Karppinen et al. (2000) presented a simplified solution for including the basic reactions of nitrogen oxides, oxygen, and ozone to a Gaussian plume model; this system of equations can be solved analytically. The chemical transformation equations were included in the dispersion model based on the so-called receptor-oriented discrete parcel method (Kukkonen et al., 2001a, Kukkonen et al., 2001b; Karppinen et al., 1998). This method considers air parcels, in which the emissions and background air are assumed to be instantaneously uniformly mixed. The chemical reactions in each parcel are then assumed to proceed independently of the dispersion process.

Regarding the transformation of nitrogen oxides, the influence of hydrocarbons is important on regional and long-range transport scales, but commonly insignificant on the urban scales. However, their influence may be substantial in episodic conditions, during prevailing stable atmospheric stratification and low wind speed, and in case of recirculation of the air masses over a city.

In the present study it was assumed that most (but not all, e.g., 75% depending on emission source category) of emitted NO_x_ emissions are initially NO. There is uncertainty in the ratio of primary NO_2_ emissions, and in addition, the ratio is gradually changing caused by technological advancement. Most detailed information is available for road traffic; in (Grice et al., 2009) the ratio of primary NO_2_ to NO_x_ is estimated to be above 10% in 2005 and projected to exceed 30% by 2020 within the European Union. During the atmospheric transport NO is converted into NO_2_, and the reactions involving O_3_ are responsible for most of the oxidation. However, the conversion of NO to NO_2_ can be limited by the amount of O_3_ at the location. A solution to this is to make the model predictions at a specific order, i.e., the prediction for O_3_ at the RP is done before predicting NO_2_ and NO. Then, a post-processed adjustment can be applied to the modelled NO_2_ and NO, given that the O_3_ concentration at the RP can be given as input. Further, it is assumed that NO released this way is immediately converted into NO_2_, simultaneously depleting O_3_ through oxidation by introducing a modelled virtual negative O_3_ emission. In this step we base the conversions on the molecular masses of the species involved. After the assessment of Eq. (1) it is possible to check whether the initial assumptions were correct and if not, balance the modelled NO, NO_2_ and O_3_ so that O_3_ is non-negative. As an example, near major roads a large NO_2_ concentration is initially modelled coupled with a negative (virtual) total O_3_ concentration. After the balancing measure some part of the modelled NO_2_ is transformed back to NO.

Finally, to address the atmospheric lifetime of NO_2_ mass decay functions are used. For instance, according to (Liu et al., 2016) the mean lifetime for NO_2_ (power plants, May–September, USA and China) was derived to be approximately 4 h and based on this 30% of the NO_2_ mass is reduced each hour. Such decay functions are only applied to Gaussian puff modelling, in which the state and movement of the emission parcels are updated dynamically.

### 2.4 Data assimilation

The data assimilation method presented in this paper variates the hourly emission factors for the included emission source categories, effectively making the modelling to be constrained by the observations. In addition to emission factors, there are other explanations for having differences between measured and modelled observations; these include meteorological, chemistry or dispersion modelling-related reasons but these are not considered in the data assimilation.

A model prediction for any pollutant species given by Eq. (1) can be expressed as the sum of its category-specific contributions, since emission source category distinction is maintained for both the Gaussian Puff and Plume modelling: $C\left(x,t\right)=\underset{s\;\in \;S}{\sum }c_{s}\left(x,t\right),$ for a total of S emission source categories in which the regional background has been included. The term $c_{s}\left(x,t\right)$, for example, stands for concentrations caused by traffic, shipping or RWC at location x during time t. An adjusted model prediction, that is affected by recent and historic data assimilation results, is given by:

(5) $$\begin{array}{l} C^{'}\left(x,a,t\right)=\sum_{s\;\in \;S}c_{s}^{'}\left(x,t\right),\;\\ c_{s}^{'}\left(x,t\right)=a_{s}A_{s}c_{s}\left(x,t\right),\;s\neq BG,\\ c_{BG}^{'}\left(x,t\right)=a_{BG}+A_{BG}+\;c_{BG}\left(x,t\right),\;c_{BG}^{'}\geq 0 \end{array}$$

where $a\;=\;\left[a_{1},\;a_{2},..a_{S}\right]$ is a set of hourly adjustments for emission source categories, and $A_{s}$ is the persistent adjustment for category S. The term ‘persistent adjustment’ is used, since $A_{s}$ encapsulates the outcome of many previous hourly adjustments, and its effect to Eq. (5) ‘persists’ even when no measurement data is available (e.g., during forecasting). In such cases when no measurement data is available $a_{s}=1$, $\;a_{BG}=0$.

The hourly adjustment set a is optimized during data assimilation as a free parameter - for each modelling hour and separately for each pollutant species. The value of $A_{s}$ is considered constant for the estimation of Eq. (5), but each optimized $a_{s}$ will slightly update and modify the value of $A_{s}$ for subsequent modelling hours. This update operation also considers the time-of-day and the day-of-week, i.e., if an hourly adjustment $a_{s}$ for a particular local time is consistently high or consistently low, then the persistent adjustment $A_{s}$ for that local time gradually adapts to match this behaviour. More information is given in supplements.

The weighted squared error (WSE) between an adjusted model prediction and the observation $o_{m}\left(t\right)$ is $w_{m}{\left[o_{m}\left(t\right)\;–\;C^{\prime }\left(x_{m},a,t\right)\right]}^{2}$, where the weight factor $w_{m}$ relates to the measurements m reliability and variability. The aim of the data assimilation is to minimize the weighted sum of squared errors (WSSE) across all the measurements for the same pollutant species at a given hour. This formulation is like the one in weighted least squares technique (WLS) (Kiers, 1997). In WLS, in which the best linear unbiased estimator is solved to minimize WSSE, the inverse variance of the measurement is often used for $w_{m}$ ($w_{m}\;=1/\sigma^{2}$). In this approach $\sigma^{2}$ is being estimated based on a fixed quality rating for the measurement device considering the hourly variability of measurements from the measurement source (Johansson et al., 2015). The WLS technique, however, is not adopted for solving in ENFUSER; strict limitations for possible states for a need to be enforced, e.g., minimum and maximum values for $a_{s}$. The system due to Eq. (5) is not easily expressed as linear expressions either. Further, due to meteorological conditions, infrequent calibration, and technical issues, $w_{m}$ needs to be treated as a free parameter that is also optimized during data assimilation.

For M measurements (in M locations for a certain pollutant species) the optimization problem for minimizing the WSSE is defined as follows:

(6) $$min\left\{WSSE\left(a,p\right),\;WSSE\left(a,p\right)=\;f\left(p\right)\overset{M}{\underset{m=1}{\sum }}p_{m}w_{m}{\left[o_{m}\left(t\right)\;–\;C^{\prime }\left(x_{m},\;a,\;t\right)\right]}^{2}\right\}$$

where $p=\;\left[p_{1},p_{2},..p_{M}\right]$, $p_{m}\in \left[0,\;1\right]$ is a factor that reduces the weight assigned to the WSE for measurement m and f(p) is a penalty function (Smith and Coit David, 1996) that increases WSSE for each $p_{m}$ < 1. The function f(p) acts as a countermeasure when the values of $p_{m}$ are being reduced; any attempt to minimize WSSE(a,p) without incorporating a penalty function would include the trivial minimization of the values of $p_{m}$. An effective penalty function forces the algorithm to search for a balanced compromise of optimal adjustments to emission factors (***a***), while keeping $p_{m}$ collectively as high (close to 1) as possible. With this in mind we define:

(7) $$f\left(p\right)=\sum {p_{m}}^{-b}$$

where a value of 1 is used for $b,\;\left(b>0\right)$. Several other formulations for $f\left(p\right)$ have been tested; the suggested one is currently the simplest well-performing parametrization found based on numerical test simulations.

#### 2.4.1 Discrete descent algorithm for searching the optimum

The optimization task of Eq. (6) is a nonlinear optimization problem (Ruszczynski, 2011). The problem can be simplified by discretizing the variables; however, no completely satisfactory method exists to solve it even in that case (Olsen and Vanderplaats, 1989). Gradient descent methods for which an overview is presented in (Ruder, 2016) are one of the more popular approaches to perform this kind of optimization. The logic is simple: one can slide towards the minimum by moving to the opposite direction of the gradient of the objective function step by step. In case the system is convex the algorithm terminates at a global minimum, otherwise the potential issue of getting trapped in a local minimum must be considered.

**Fig. 6:**
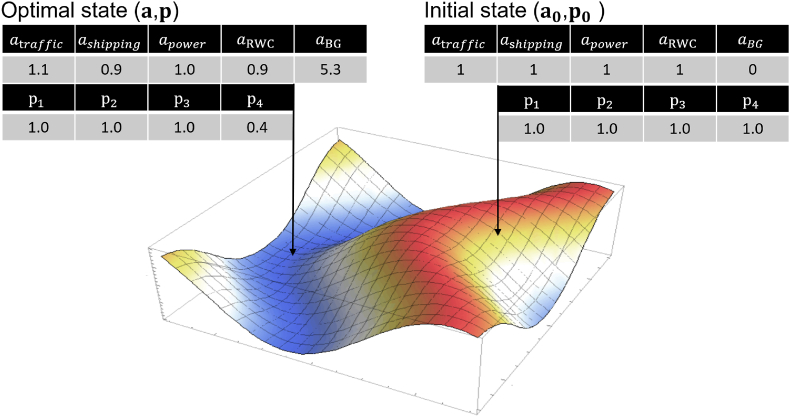
Illustration of the discrete descent algorithm with four measurements. All values are illustrative. Starting from an initial, unadjusted state, the iterations proceed to find directions in which the rate of improvement is highest, and then take a step in that direction. When there are no directions that provide improvements, the iteration terminates at a final state. Example mesh provided by Wolfram Alpha LLC. 2021. Wolfram|Alpha. http://www.wolframalpha.com/input/?i=2%2B2 (access Oct 28, 2021).

A discrete gradient descent algorithm is used in ENFUSER to find optimal parametrization for (a,p) that minimizes WSSE, starting from an arbitrary initial state (Fig. 6) and step by step descending to optimum in terms of WSSE. The possible states for each $a_{s},p_{m}$ have been discretized. For emission source adjustments, each $a_{s}$ are allowed to have a finite number of states, e.g., from 0.5 to 1.5 with a step size of 0.1. The discretized possible states for each $p_{m}$ include [1, 0.6, 0.4, 0.2, 0.1, 0.05].

In the descent algorithm the direction and step length offering the most impactful reduction to WSSE are searched and taken. Once there are no directions left to reduce WSSE the algorithm terminates. To address the potential issue of terminating at a local minimum the following techniques are used: first, attempts are made to leap over the minimum with significantly larger step sizes when the algorithm is about to terminate. Secondly, the iteration can be repeated using multiple different initial states, and then select the optimal state out from several candidates. The optimal state a that minimized WSSE is used to update $A_{s}$ (procedure has been described in supplements). The computational burden of the data assimilation algorithm can be considered negligible with respect to the Gaussian dispersion computations, even with a high number (M >100) of measurement data being included. Therefore, the method supports the use of AQ sensors in large quantities.

## 3 Results

The model was applied for the prediction of hourly pollutant concentrations for NO_2_, O_3_, PM_2.5_ and PM_10_ in the HMA during 2017 and 2018. Depending on pollutant species there are up to 12 AQ measurement stations in HMA, of which several are relocated each year. All model configuration parameters, such as the minimum wind speed and minimum mixing layer height, have been set as presented in the methods section with appendices.

**Fig. 7:**
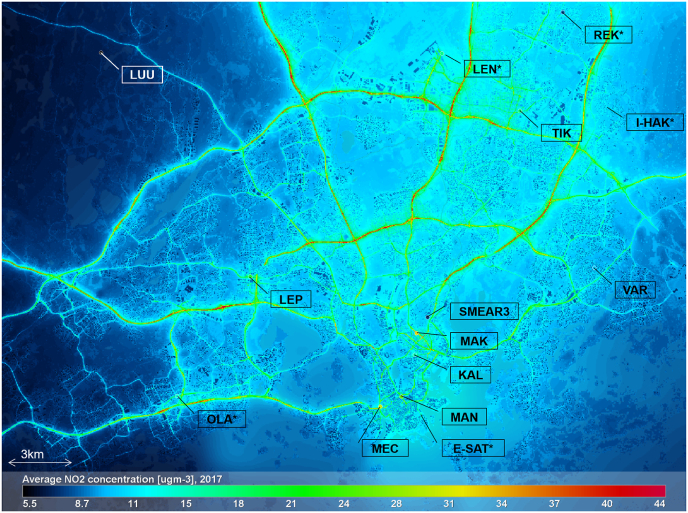
Predicted annual average NO_2_ concentrations in HMA for 2017, and AQ measurement station locations. Stations marked with ‘*’ are transferable stations that have provided measurements for this study in either 2017 or 2018. The stations have been further described in Table 2.

The station locations are shown in Fig. 7, together with annual average concentrations for NO_2_ (2017). The geographical annual averages presented in this study have been averaged from modelled hourly concentrations for the same area; further, the modelling of each hour involves urban scale Gaussian plume modelling, minute-by-minute Gaussian puff modelling, incorporation of regional background and data assimilation. The annual computations have been performed using the operational system architecture, i.e., proceeding chronologically from January to December in 24-h modelling task batches. Completing a modelling task that covers a full year this way, takes approximately three days of processing time with a modern^3^ PC.

The contribution of road traffic is dominant for NO_2_ in HMA but the concentrations near main roads are often limited by the availability of O_3_. The regional background is approx. 7 μgm^−3^ across the modelling area and shipping contributes up to 5 μgm^−3^. The contribution of power plants is minor, also for other pollutant species than NO_2_.

**Fig. 8:**
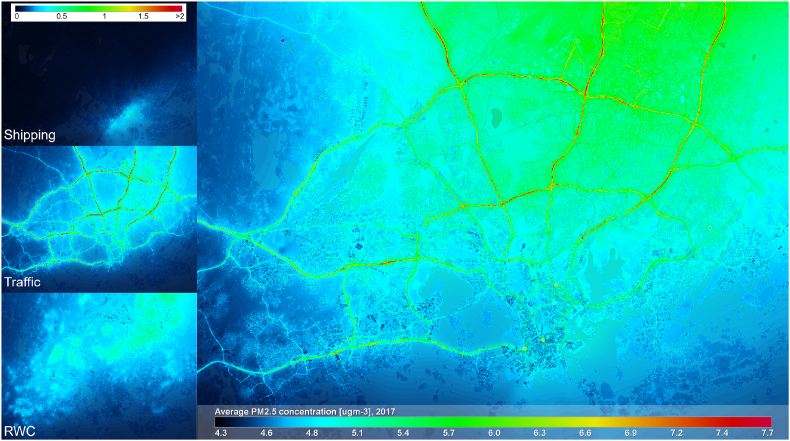
Predicted annual average PM2.5 concentrations in HMA for 2017. On the left, emission source contributions from shipping, road traffic and residential wood combustion (RWC) are shown separately on the scale of [0, 2] μgm-3.

**Fig. 9:**
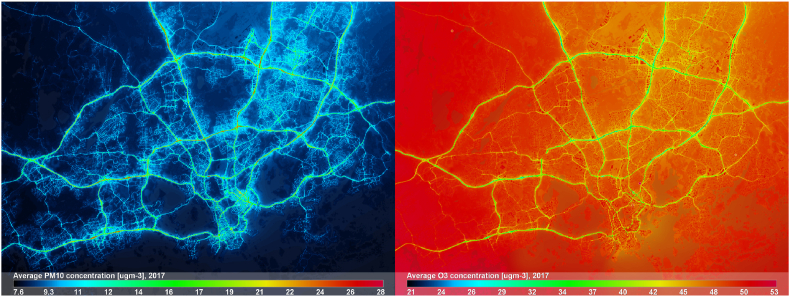
Predicted annual average PM_10_ (on the left), and O_3_ (on the right) concentrations in HMA for 2017.

In Fig. 8 the annual average PM_2.5_ concentration for 2017 is shown with emission source contributions. The most notable source is the regional background (not shown), approx. 4 μgm^−3^ across the modelling area. The contribution of traffic is up to 2 μgm^−3^ near the busiest roads in HMA; the modelled contribution of RWC emissions is lower than 1 μgm^−3^. The contribution of shipping for PM_2.5_ in 2017 is the highest near the Helsinki central area coastline, but lower than 0.5 μgm^−3^. The corresponding annual modelled average concentrations for PM_10_ and O_3_ are shown in Fig. 9. The annual average concentrations of PM_10_ and PM_2.5_ are naturally related, the most notable difference being the added resuspension of particles that increases concentrations near roads. For O_3_ there is no other source for pollution other than the regional background, however, lower annual average O_3_ concentrations can be observed nearby traffic and shipping emission sources due to adopted NO_x_ - O_3_ chemistry.

### 3.1 The evaluation of the model against measured data

The model evaluation was performed using the leave-one-out cross-validation procedure. For each measurement site, the model predictions were done excluding the values at that site during data assimilation. Due to model development, measurement availability and model maintenance during 2017 and 2018 the evaluation time series does not cover a full two-year period and there are some notable gaps in between. The measurements used for the evaluation are uncorrected, real-time data extracted and archived as soon they have become available online. This means that the air quality measurements have not yet been subject to the more stringent QA/QC procedures and may contain outlier data. For most stations participating in the evaluation there are more than 7500 hourly measurements available for comparison annually. For all sites a measurement height of 3m above ground has been assumed, using the exact coordinates as shown in Table 2. In this study, several standardized statistical measures were used to quantify the modelling performance; these measures have been explained in the supplements.

**Fig. 10:**
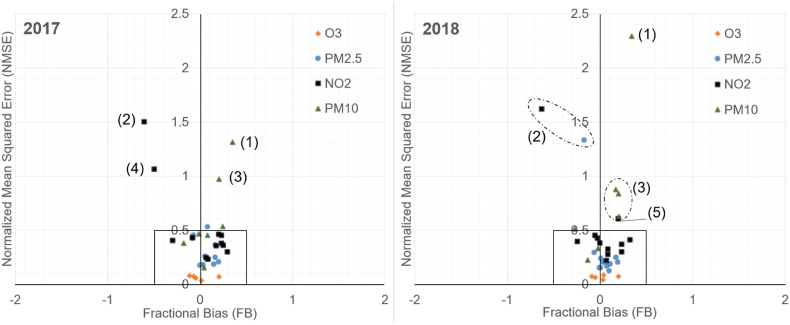
A map of NMSE and FB for all stations used for the evaluation in 2017 and 2018. The box near the origin illustrates the target values for these two metrics. The outlier stations outside this target box have been grouped into 5 outlier cases, for which further details have been given in the discussion section.

In terms of fractional bias (FB) and normalized mean squared error (NMSE) the model prediction accuracy has been presented in Fig. 10 for each station and species for 2017 and 2018. Further details have been shown in Table 1, Table 2. As it can be seen from Fig. 10, for O_3_ and PM_2.5_ most of the stations are within the target limits with respect to NMSE and FB (NMSE <0.5 and −0.5< FB < 0.5.), however for NO_2_ and PM_10_ there are a couple of outlier stations that cause the NMSE and F2 to exceed these target values.

**Table 1:** Evaluation results for 2017 (upper) and 2018 (lower) for individual measurement stations. For all stations and pollutant species the following metrics are shown: F2 = Factor-of-two, PCC = Pearson correlation coefficient (r), NMSE = normalized mean squared error, FB = fractional bias. The formulas used for the metrics have been defined in supplements.

Station	NO_2_				PM_2.5_				O_3_				PM_10_			
	F2	PCC	NMSE	FB	F2	PCC	NMSE	FB	F2	PCC	NMSE	FB	F2	PCC	NMSE	FB
**2017**																

**VAR**	0.78	0.68	0.43	−0.08	0.87	0.76	0.26	0.05	0.90	0.85	0.06	−0.05				
**REK**	0.74	0.75	0.41	−0.30	0.77	0.51	0.43	−0.08								
**KAL**	0.86	0.74	0.36	0.17	0.85	0.84	0.18	0.00	0.94	0.90	0.04	0.01	0.92	0.87	0.16	0.04
**OLA**	0.83	0.72	0.46	0.20	0.87	0.81	0.21	0.20					0.88	0.71	0.54	0.25
**LEP**	0.84	0.76	0.38	0.23	0.86	0.60	0.53	0.08					0.84	0.71	0.97	0.20
**LUU**	0.47	0.67	1.50	−0.60	0.73	0.66	0.45	−0.07	0.89	0.82	0.07	−0.07				
**MAK**	0.82	0.80	0.30	0.30	0.88	0.84	0.19	0.15	0.87	0.87	0.08	−0.11	0.84	0.75	1.32	0.35
**MEC**	0.89	0.73	0.25	0.06	0.86	0.70	0.25	0.16					0.82	0.78	0.38	−0.18
**TIK**	0.85	0.79	0.36	0.25	0.88	0.80	0.25	0.06					0.89	0.79	0.46	0.08
**SMEAR3**	0.60	0.41	1.07	−0.50					0.93	0.91	0.07	0.21				
**LEN**	0.70	0.61	0.45	0.23	0.86	0.74	0.18	0.03								
**MAN**	0.88	0.72	0.23	0.08	0.81	0.67	0.36	0.16					0.83	0.69	0.47	−0.01
**Averaged**	0.77	0.70	0.52		0.84	0.72	0.30		0.91	0.87	0.06		0.86	0.76	0.61	
**2018**																
**VAR**	0.74	0.70	0.45	−0.06	0.92	0.84	0.17	0.07	0.90	0.84	0.07	−0.05				
**KAL**	0.85	0.75	0.33	0.08	0.87	0.85	0.15	0.01	0.93	0.88	0.04	0.03	0.89	0.83	0.22	−0.13
**KAU**	0.79	0.74	0.38	0.00	0.91	0.83	0.19	0.11					0.91	0.70	0.88	0.17
**LEP**	0.82	0.75	0.37	0.23	0.91	0.73	0.24	0.01					0.84	0.60	2.29	0.34
**LUU**	0.51	0.68	1.62	−0.63	0.77	0.35	1.34	−0.17	0.87	0.80	0.09	0.04				
**MAK**	0.84	0.76	0.30	0.24	0.92	0.87	0.13	0.10	0.88	0.87	0.07	−0.09	0.81	0.68	0.84	0.20
**MAN**	0.87	0.74	0.22	0.07	0.82	0.70	0.25	0.17					0.83	0.69	0.33	−0.02
**MEC**	0.86	0.70	0.28	0.09	0.89	0.82	0.20	0.19					0.73	0.71	0.51	−0.27
**TIK**	0.80	0.78	0.41	0.33	0.90	0.81	0.20	0.03					0.87	0.80	0.63	0.20
**SMEAR3**	0.78	0.74	0.40	−0.24					0.93	0.90	0.07	0.20				
**E-SAT**	0.71	0.61	0.61	0.20	0.87	0.84	0.15	−0.01								
**I-HAK**	0.79	0.72	0.43	−0.03	0.81	0.66	0.30	−0.06								

**Averaged**	0.78	0.72	0.48		0.87	0.75	0.30		0.90	0.86	0.07		0.84	0.71	0.81

**Table 2:** Station coordinates, data point count (based on NO_2_) and annual average predictions (Pred.) against annual average observed concentrations (Ob.) are shown. Results are shown separately for 2017 (upper table) and 2018 (lower table). In the station description ‘bgr.’ stands for ‘background’, ‘h.’ for ‘housing’ and ‘streetc.’ is used for ‘street canyon’.

					NO_2_		PM_2.5_		O_3_		PM_10_	
Station	Latitude	Longitude	Description	Count	Pred.	Ob.	Pred.	Ob.	Pred.	Ob.	Pred.	Ob.
**2017**												

**VAR**	60.22393	25.10244	detached h. area	7805	12.0	11.0	5.3	5.6	49.0	46.8		
**REK**	60.33142	25.07584	detached h. area	5482	11.8	8.8	5.5	5.1				
**KAL**	60.18739	24.95061	urban bgr.	7941	13.1	15.5	5.0	5.0	47.8	48.5	10.5	11.0
**OLA**	60.16997	24.74924	suburban traffic	5598	12.0	14.6	4.6	5.6			9.2	11.8
**LEP**	60.22024	24.81133	urban traffic	7903	16.3	20.6	5.3	5.8			11.6	14.3
**LUU**	60.31439	24.68486	rural bgr.	7873	8.1	4.4	4.9	4.5	52.6	48.9		
**MAK**	60.19644	24.95198	urban streetc.	7915	24.5	33.0	5.5	6.4	42.7	38.1	13.7	19.5
**MEC**	60.16558	24.92142	urban traffic	5648	29.3	31.3	5.5	6.5			20.0	16.7
**TIK**	60.28995	25.03953	urban traffic	7826	14.3	18.4	5.6	5.9			10.1	10.9
**SMEAR3**	60.20307	24.96131	urban bgr.	5856	13.2	7.9			46.2	56.9		
**LEN**	60.31406	24.97359	airport traffic	5531	13.9	17.5	5.4	5.6				
**MAN**	60.16964	24.93924	urban streetc.	7980	24.7	26.8	5.4	6.4			18.9	18.6
**2018**												
**VAR**	60.22393	25.10244	detached h. area	7291	11.7	11.1	6.6	7.1	53.3	50.7		
**KAL**	60.18739	24.95061	urban bgr.	7441	14.4	15.7	6.5	6.6	50.5	52.1	13.8	12.0
**KAU**	60.20957	24.72994	suburban traffic	7707	15.2	15.2	6.5	7.2			14.8	17.5
**LEP**	60.22024	24.81133	urban traffic	7647	17.6	22.3	7.0	7.1			14.6	20.6
**LUU**	60.31439	24.68486	rural bgr.	7681	10.9	5.7	6.9	5.8	51.2	53.4		
**MAK**	60.19644	24.95198	urban streetc.	7948	24.9	31.6	7.0	7.7	45.8	41.9	16.8	20.6
**MAN**	60.16964	24.93924	urban streetc.	7227	26.6	28.6	6.9	8.2			24.3	23.8
**MEC**	60.16558	24.92142	urban traffic	7676	29.7	32.4	7.2	8.7			25.6	19.5
**TIK**	60.2900	25.03953	urban traffic	7646	14.8	20.5	7.1	7.3			12.9	15.8
**SMEAR3**	60.20307	24.96131	urban bgr.	7692	13.7	10.7			49.7	60.9		
**E-SAT**	60.16221	24.95686	urban ship term.	7199	13.2	16.1	6.0	5.9				
**I-HAK**	60.29175	25.11242	detached h. area	7656	12.0	11.6	6.5	6.1				

**Fig. 11:**
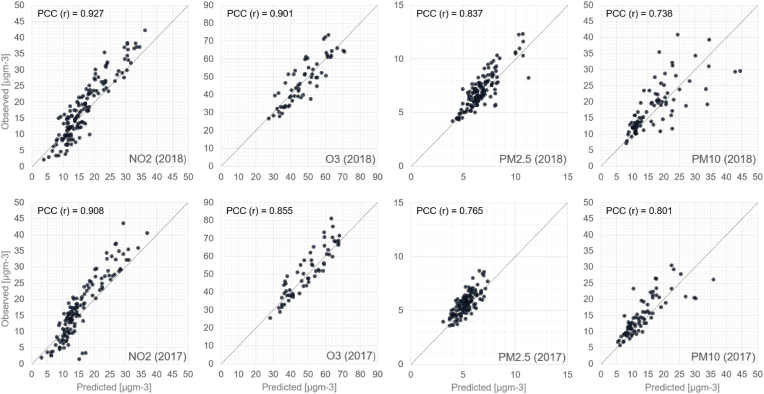
A comparison of average monthly predicted (horizontal axis) pollutant concentrations against measured (vertical axis) averages for NO_2_, O3, PM_2.5_ and PM_10_, for both evaluation years of 2017 and 2018. For each plot the Pearson correlation coefficient (r) has been shown at the top left corner. A data point in the scatter plot represents a monthly average (predicted and measured) for a single station; the data of all available stations have been included in the plots.

An evaluation of predicted monthly averages against observed ones have been shown in Fig. 11. In terms of correlation (PCC) the highest ones are obtained for NO_2_, 0.908 for 2017 and 0.927 for 2018. The second highest correlation is obtained for O_3_.

Previous deterministic model evaluations for PM_2.5_ in HMA have been presented by Kukkonen et al. (2018 and 2020) for the years 2004–2014. Most of the stations are the same as those used in this study. Over this assessment period the average F2 was approximately 0.68 and the index of agreement (IA) was 0.66 for the deterministic modelling system. For comparison, the average ENFUSER predictions (for 2017 and 2018) for F2 were 0.84 and for IA these were 0.83. As expected, the evaluation measures computed in this study are better than the corresponding ones obtained using solely deterministic methods.

## 4 Discussion

The spatial distribution of annual average concentrations for 2017 was presented in the study. The corresponding annual averages for 2018 were not shown, as these are like the ones obtained for 2017.

Several outlier stations can be observed in Fig. 10 when model predictions are compared to measurements in terms of NMSE and FB. These outlier stations have been grouped as follows: Outlier 1 stands for an urban traffic station (LEP) situated in between a busy outdoor parking area and a road. For this site the model underpredicts PM_10_ concentrations, especially during spring road dust events.

Outlier 2 is a rural background station (LUU) that provides valuable information for the model for regional background concentrations. The added value comes from the fact that the model predictions at this rural background station can effectively be variated by adjusting $a_{BG}$, and the model prediction at LUU is not sensitive to other $a_{s}$. When this measurement data source is being omitted in the leave-one-out validation procedure, a collection of suburban, and urban stations remains. From the data assimilation method's perspective, after the omission of LUU there is no measurement data available to provide a pure signal for the regional background. Further, for NO_2_ the modelling results for LUU are the poorest; a clear over-prediction can be observed especially when the wind direction is from South and South-East. With these wind directions the Gaussian puff modelling carries NO_2_ pollution to LUU, which in turn suggests that the used decay rate for NO_2_ may be underestimated.

Outlier group 3 is a collection of challenging urban/suburban locations for PM_10_ modelling, of which the hardest one to predict is an urban street canyon station (MAK). For this group the NMSE target is exceeded but the F2 and FB targets are met. Nearby construction work has also been suggested as one of the causes for high measured PM_10_ concentrations.

**Fig. 12:**
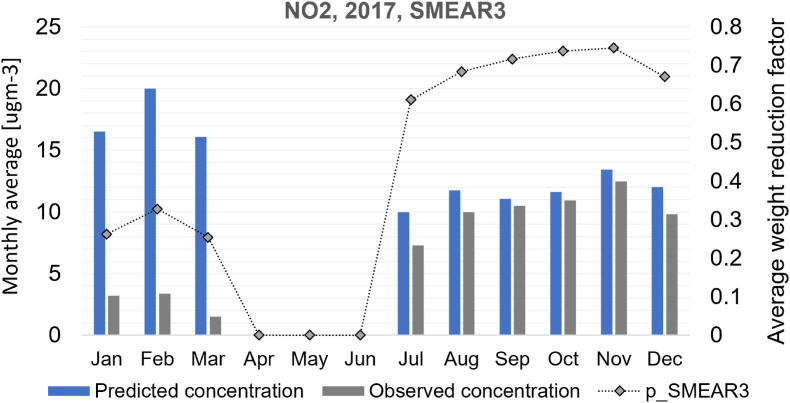
Average predicted and observed monthly NO_2_ concentration at SMEAR3 station (2017), and the average weight reduction factor ($p_{m}$) assigned for SMEAR3 during data assimilation. All twelve NO_2_ stations were included in data assimilation, including SMEAR3.

Outlier 4 is an urban background station (SMEAR3), which provided very low concentration measurements for NO_2_ from January to March 2017. These measurements were later confirmed to be erroneous by the data provider. After mid-July this station returned online after being offline for 3 months. This kind of conflicting measurement input (with respect to other measurement evidence), however, can be dealt with by the data assimilation method as is shown in Fig. 12. Monthly average concentrations and average weight reduction factors for Eq. (6) are shown for the SMEAR3 station in the figure, in a modelling test run where every station participates in data assimilation. In this modelling test from January to March, the data assimilation algorithm often terminates at states that strongly reduce the weight put on SMEAR3 measurements. The only effective way to reduce the squared error (SE) at SMEAR3 would be to reduce $a_{BG}$, however, such an adjustment would increase SE in all other measurement locations simultaneously. Therefore, a reduction of $a_{BG}$ does not occur at the optimum where data assimilation terminates, but a reduction of weight put to the erroneous SMEAR3 measurement occurs instead.

Outlier 5 is a coastal measurement site near a shipping terminal (E-SAT). Particularly the prediction of hourly NO_2_ is challenging there, especially due to berthing ships nearby for which the emissions of auxiliary engines are difficult to predict by the STEAM3-model. The road traffic profiles near E-SAT seem also to be underestimated, as they do not account for the additional traffic induced by the nearby shipping terminals. Similarly, in 2017 one of the measurement stations was situated at the Helsinki Airport (LEN). While NMSE and FB are acceptable for this location, the model clearly under-estimates NO_2_. One possible explanation for this is the significant amount of taxi traffic (with mostly diesel engines being used) nearby the station, which has not been accounted for in the vehicular traffic flow mappings in the area.

In Fig. 11 the predicted monthly average concentrations against measured concentrations were shown. For 2018 high correlation of 0.837 is obtained for PM_2.5_ but a lower correlation is obtained for 2017. This is likely due to higher monthly variability for the average PM_2.5_ concentrations in 2018 and this variability is successfully being captured by the model. Between January and March 2018, the average temperature is more than 3 °C lower than in 2017, which in turn results in higher emission output from the residential wood combustion sources during the winter months. The lowest correlations are obtained for PM_10_. Especially during Spring 2018 the monthly variability is strong and there are notable differences from station to station due to road dust.

In HMA the modelled regional background concentrations (as given by the CTM) for ozone have a clear seasonal bias; during Spring the concentrations are underestimated by the CTM. The adopted data assimilation procedure can correct the regional background to more appropriate levels. Namely, $A_{BG}$ for ozone begins to rise during Spring, reaching 5 μgm^−3^. After Summer $A_{BG}$ reduces gradually to a value close to 0 μgm^−3^. For other modelled pollutant species such a distinguished seasonal behaviour is not observed. For example, $A_{BG}$ for NO_2_ varies in between −1.5 and 0.5 μgm^−3^ throughout 2017 and 2018 without clear seasonal patterns.

Occasional overfitting by the data assimilation procedure can also be observed for several pollutant species. During winter months NO_2_ traffic emissions are being scaled upwards and downwards during summer; modelled PM_10_ background is being increased by PA during spring-time road dust episodes. Fortunately, these occurrences of overfitting can be analysed and used to guide further model improvement. For example, the adjustments made by the data assimilation method can be analysed in terms of conditional averages, i.e., as a function of meteorological variables such as wind speed, boundary layer height and ambient temperature. If the data assimilation systematically assigns strong adjustments in specific atmospheric conditions, then this is a clear indication of overfitting.

### 4.1 Limitations

The key sources of GIS information are extracted from OpenStreetMap, however, the quality of this information varies between countries; in some areas, such as in Asia, additional supplemental GIS information may be needed. Further, information in addition to the GIS data is commonly needed to reliably evaluate traffic flow patterns. The model has limited capability to address atmospheric chemistry; the model currently includes solely a simplified scheme for the NO_x_ - ozone chemistry. A more comprehensive treatment of the chemical and physical transformations would be needed for geographically more extensive modelling domains, and in case of more complex meso- and microscale meteorological processes (e.g., recirculation of air masses over a city).

While the data assimilation method provides a mechanism for potentially improving the emission strengths of the local emission modelling, the method cannot correct any spatial, heterogeneous inaccuracies within the used emission inventories. The model also cannot explicitly correct for any inaccuracies in the dispersion modelling and the evaluation of other properties of the emission sources, for instance, the release height estimated for the emissions from residential wood combustion. The modelling can in some cases result in an over-fitting to the measured air quality data, due to the adopted data assimilation procedure. For example, during spring road dust episodes the modelling may excessively increase the regional background concentrations, to counterbalance the lack of sufficiently realistic modelling for dust accumulation and resuspension.

In addition, the modelling requires the data of an air quality measurement network in the modelling area. The data assimilation method can function effectively only if this measurement network includes a wide range of stations in different environments. Accurate coordinates and measurement heights are also needed for each station or sensor, which will be included in the data assimilation. The Gaussian dispersion modelling methods cannot address explicitly either the effects of buildings and other obstacles, or the effects of complex terrain (such as mountainous areas, valleys, and fjords). The modelling has applied simple methods for estimating Gaussian standard deviations, atmospheric stability classes and urban micrometeorology, and upwind roughness length. These simplifications were needed partly due to the computational efficiency requirements and the need to support offline pre-computations for Gaussian dispersion modelling.

### 4.2 Future development topics

The modelling of PM_10_ could be improved in the future, especially regarding the modelling of road dust during spring. This development work could include the use of machine learning methods (neural networks or random forest models). Machine learning methods may also be adopted in the future to assist in the modelling of traffic flow patterns within the OSM-road network, provided that enough traffic flow measurements will be available for training such models. Detailed traffic flow datasets, such as the ones used in the Helsinki Metropolitan Area, may not be available for other urban areas.

The operational modelling service for HMA will be improved by extending the forecasting period up to 24h in the future. Black carbon and the lung deposited surface area (LDSA) of particles will be included in the modelling; measurement data is available for both of these pollutant species in the Helsinki region. In addition, an existing complementary network of AQ sensors will be utilized. Finally, the model could continuously use the predicted near-real-time shipping emissions instead of historic annual emission data. To facilitate this, the STEAM3 model will be coupled with a continuous supply of AIS data for the modelling area.

## 5 Conclusions

We have presented a multi-scale air quality modelling system called ENFUSER. The model uses a combination of Gaussian plume and puff modelling techniques, incorporates regional scale modelling, and utilizes data fusion and AQ measurement -based data assimilation. This novel modelling system has provided open-access information on air quality for the general public continuously since March 2018.

Clearly, the model can be used also for other regions in addition to the HMA. The model takes advantage of both a global, open-access source for GIS information and various time-dependent dynamic input datasets. The extracted GIS data and the time-dependent datasets are used in the model in various ways, including a), the refinement and downscaling of emission inventories b), description of urban buildings and other obstacles for local dispersion modelling c), the assessment of surface properties for the processing of meteorological information and d), the detailed modelling of vehicular flow patterns and the emissions of road traffic. The model can in some cases be used with reasonable accuracy even in regions, for which reliable emission inventories are not available, due to the data fusion and data assimilation capabilities. However, some information sources used in this study are not available at all locations internationally.

To guarantee the transferability of the model to another city than Helsinki, the following key datasets and sources of information should be available. First, there should be an AQ measurement network that provides measurements for each modelled pollutant species. There should be several air quality measuring stations, and preferably the stations should be distributed in different measurement environments (e.g., one or more in rural background areas and several in urban areas). In case the number of stations in the area is low, a complementary network of sensors may be needed. If the measurement data is not accessible from global open-source data portals, then a timely access to the measurement data needs to be provided. Second, there should be information available to support the customization of traffic flow patterns in the road network, possibly in the form of a previous study or a collection of traffic count data. Third, in case there are special local emission sources in the area (e.g., industrial areas) that are known to substantially affect the air quality, then emission inventories for these sources should be provided. Fourth, the current provider of HARMONIE NWP data for ENFUSER covers only the Baltic Sea region, and a different source for meteorological input is needed elsewhere. In addition to these necessary sources of information, there are certain lower priority datasets that may be provided to improve the modelling quality but are not considered mandatory. For example, a building height dataset improves the modelling capabilities in view of urban micrometeorology and can be used to address the concentrations in urban street canyons.

A novel data assimilation method was presented to support urban scale modelling. This method variates the local emission factors under the constraints of the measured air quality information. The method can continuously adjust the modelling of emissions over time; it will adapt to the changes in the temporal patterns of emissions and the regional background concentration. The method is computationally efficient and can be used for the simultaneous assimilation of large amounts of measurement data.

The model was evaluated in this study in a now-casting mode, using a two-year evaluation dataset for the Helsinki Metropolitan Area during 2017 and 2018. The modelling accuracy can be considered good. In terms of monthly averages, there was a high correlation (PCC) for the predicted and measured concentrations for NO_2_ (0.908 and 0.927) and O_3_ (0.855 and 0.901) for both years. In the case of PM_2.5_, the achieved correlation for 2018 was 0.837. Although the correlation for monthly averages for NO_2_ was high, the model on the average systematically overestimated the relatively lower measured NO_2_ concentrations and underestimated the higher NO_2_ concentrations. In the case of the PM_10_ concentrations, there was a relatively lower correlation (PCC) in terms of monthly averages for both years (0.801 and 0.738). In terms of hourly evaluation and specifically in terms of fractional bias and normalized mean squared error, the best modelling performance was obtained for PM_2.5_ and O_3_, while the model performance measured for PM_10_ was relatively worse. The model performance measures were on the average clearly better compared with those found in previous studies in this region, using deterministic modelling without data assimilation.

## Author contributions

LJ is the lead author of the article and the developer of the ENFUSER model. LJ also prepared results using the model. Ari K, MK, JN, Anu K and JK internally reviewed and contributed to the writing of the article. JN and Anu K provided emission inventory input for the model with regard road traffic and RWC.

## Software and data availability

The ENFUSER model, developed by the lead author, has been written in Java (more than 75 000 lines of code) during the years 2012–2022. The model at its earlier stages of development has been presented in (Johansson et al., 2015). ENFUSER has been designed to be used in a modern PC (e.g., 8-core CPU and 32 GB of RAM) or in a virtual environment (both Linux and Windows are supported). The model utilizes parallel computations (multi-threading) and therefore benefits from the availability of a high number of processing cores. Data produced for the Helsinki Metropolitan Area by the model is publicly available via the Open Data portal of FMI (FMI, 2021). Source code for the ENFUSER model is publicly available via GitHub under the MIT license: https://github.com/johanssl/EnfuserMIT.git. The repository contains the necessary code and input data for operative modelling of air quality in the Helsinki region.

## Declaration of competing interest

The authors declare that they have no known competing financial interests or personal relationships that could have appeared to influence the work reported in this paper.

## Figures and Tables

**Fig. A1:**
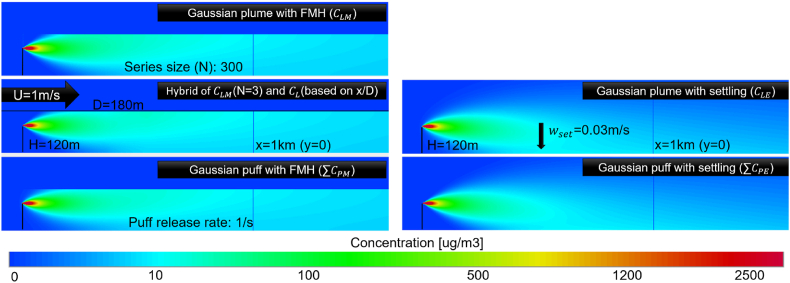
comparison of concentration fields as given by the different modelling approaches, using a single elevated unit emitter (1 g/s) as a source. In each example the vertical axis corresponds to z [0, 300m] and the horizontal axis corresponds to x (downwind distance from the emission source. Stability class ‘C’ has been used. A very high settling velocity has been used for demonstrative purposes .

**Fig. B1:**
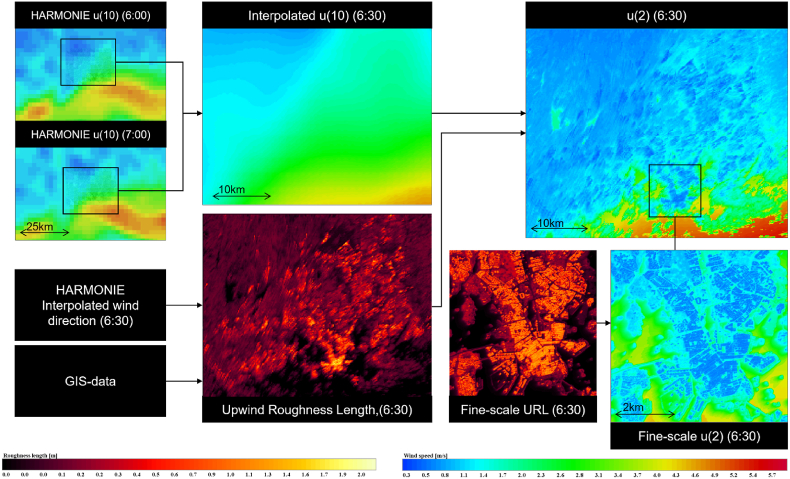
An illustration of the wind profiling methodology adopted in ENFUSER. NWP wind speed and direction are interpolated spatially and temporally and used as input. ‘6:00’ and ‘7:00’ refer to the local time of day used for the shown example for Helsinki region. Upwind roughness length (URL) estimates are produced based on the wind direction and GIS-data for the area. Wind speed profiles as a function of height are then estimated based on interpolated meteorology and URL, and this assessment is done in different scales to match the use-case they are used for. ‘u(10)’ and ‘u(2)’ refer to profiled wind speed at 10m and 2m above ground..

## Appendix A Application of dispersion modelling formulas

The extended Gaussian puff and plume formulas, that are utilized separately for gaseous species and PM_2.5_ (finite mixing height -adjusted form), and coarse particles (settling velocity -adjusted form), are presented in this appendix. The formulas and notations from (Stockie, 2011) are followed, in which variables $r_{y\;}=\;{\sigma^{2}}_{y}/2$, $r_{z\;}=\;{\sigma^{2}}_{z}/2$ and $r=\sqrt{r_{y\;}r_{z}}.$ For the expression of the extended formulas, Eq. (2) is written as:

(A1) $$c_{L}=\frac{Q}{4\pi ur}YB,\;Y=\exp\;\left(-\frac{y^{2}}{4r_{y}}\right),\;B=\exp\;\left(-\frac{{\left(z-H\right)}^{2}}{4r_{z}}\right)\;+\;\exp\;\left(-\frac{{\left(z+H\right)}^{2}}{4r_{z}}\right)$$

The Gaussian plume formula does not account for a finite mixing layer height (FMH) for the pollutant concentrations, which can range from several kilometres down to several tens of meters. Extended Gaussian plume formula that considers FMH is presented e.g., in (Stockie, 2011) and is given by:

(A2) $$c_{LM}=\frac{Q}{uD\sqrt{\pi r}}YB_{M},\;B_{M}=\left[\frac{1}{2}+\;\overset{\infty }{\underset{n=1}{\sum }}cos\;\left(\frac{n\pi z}{D}\right)\;cos\;\left(\frac{n\pi H}{D}\right)\;exp(-r_{z}{\left(\frac{n\pi }{D}\right)}^{2}\right]$$

where D is the value of FMH in meters. Low value for FMH forces the pollutant concentrations to mix into a smaller volume of air, causing larger ground-level concentrations. Due to this sensitivity of modelled concentrations with low values of FMH, a minimum value of 100m is imposed.

For assessing $B_{M}$ numerically the infinite trigonometric series needs to be cut into a finite number of N terms. The choice of N has clearly a notable impact on the computational cost of solving Eq. (A2). However, when using a low value of N (e.g., N<10) a clear bias in the concentration distribution can be seen close to the emission source. On the other hand, close by the emission source $c_{L}$ (Eq. (2)) is an accurate approximation for Eq. (A2), especially when D is large. By taking advantage of these properties, a cost-efficient solution for assessing $c_{LM}$ can be summarized as follows:

● As a function of x/D the computation method switches from $c_{L}$ to $c_{LM}$. x is the distance to the emission source along the wind direction.
  o If x/D > 2 (Case 1) assess Eq. (A2) with 3 first terms in the series
  o If x/D < 1 (Case 2) $c_{L}$ is used to approximate Eq. A2
  o Otherwise, use a linear combination of cases 1 and 2.
● In trivial cases in which the emission source and the receptor point are separated by the FMH, a value of 0 is used regardless of x/D.

This solution of combining $c_{L}$ and $c_{LM}$ cost effectively is referred to as the ‘hybrid solution’ and has been illustrated in Fig A1. It is used for Gaussian plume computations for gaseous species and PM_2.5_.

### Settling velocity

To take the effect of particle settling velocity into account the Ermak formula^4^ is used, given by:

(A3) $$c_{LE}=\frac{Q}{4\pi ur}YBe_{1},\;e_{1}=exp\;\left(-\frac{w_{set}\left(z-H\right)}{2K}-\frac{w_{set}^{2}r}{4K^{2}}\right)$$

A value of 1 m^2^/s is used for eddy diffusion coefficient K and the settling velocity $w_{set}$ is approximated with Stoke's law for typical coarse particles. The original Ermak solution supports for deposition velocity to be included, however this has been assumed to be zero. Deposition effects may still be included in the form of mass decay functions in the model.

### Gaussian puff solution extensions

Following Eq. (A1) and that the distance (d) from the receptor point to the centre of the puff is $\sqrt{{\left(x-ut\right)}^{\;2}+y^{2}}$ Eq. (4) can be written as:

(A4) $$c_{P}=\frac{Q_{m}}{8{\left(\pi r\right)}^{3/2\;}}Y_{P}B,\;Y_{P}=\;exp\left(-\frac{d^{2}}{4r_{y}}\right)$$

Considering that when D is large then $c_{L}\;\approx \;c_{LM}$ and thus $B\approx \;4\sqrt{\pi r}D^{-1}B_{M}.$ Based on this, an extended Gaussian puff solution formula that considers finite mixing height when D is large, is approximated by

(A5) $$c_{PM\;}=\frac{Q_{m}}{2\pi rD}B_{M}Y_{P}$$

It can be numerically verified that for a continuous stream of incrementally small puff releases, the use of Eq. (A5) for $c_{PM\;}$ yields a concentration field that corresponds to the field given by $c_{LM\;}$ regardless of the value of D. One such comparison has been shown in Fig A1 using D = 180m. Further, in analogy to Gaussian plume computations the Gaussian puff computations can also be performed using a cost-effective hybrid solution; the concentrations for puffs are computed based on Eq. (A5) only when x/D is low (x corresponding to the travel distance of the puff). Finally, the Gaussian puff solution formula that considers particle settling velocity is given by:

(A6) $$c_{PE}=\frac{Q_{m}}{8{\left(\pi r\right)}^{3/2\;}}Y_{P}Be_{1}$$

## Appendix B wind profiling

The available meteorological input for the model is HARMONE NWP hourly data with a resolution of 1 × 1 km for the height 10m above ground. This data is currently available only for the Baltic Sea region and in other modelling areas a different (often with lower resolution) is used. For Gaussian modelling in ENFUSER (for both plume- and puff modelling) the meteorological input is downscaled to match the needs of the multi-scaled modelling approach, and this process has been outlined in Fig B1.

The first stage of the downscaling process is to obtain an interpolated wind speed and direction values for a given location at a given time based on the NWP data. These are obtained with spatial-temporal interpolation from the raw NWP data; spatially an inverse distance -weighted kriging is used, a linear interpolation is used for time. Turning of wind direction as a function of height is not considered.

The second stage involves the estimation of upwind roughness length (URL, or $Z_{eff\;}$). The assessment needs to be wind directed, as measured turbulent fluxes can be assumed to depend on an upwind source area at the typical modelling heights used in urban scale modelling (Schmid and Oke, 1990; Schmid, 1994; Rooney, 2001). Like the approach presented in (Hammond et al., 2012), an upwind fetch distance of 500m is used and $Z_{eff\;}$ assessed this way is taken as the average value of multiple roughness length estimates ($Z_{i\;}$) encountered within the defined area of assessment. The area of assessment is set to be a narrow cone (6°) covering the area upwind up to the selected maximum distance of 500m. The distance of each $Z_{i\;}$ for the location of $Z_{eff\;}$ is considered; we estimate $Z_{eff\;}$ as a simplistic distance weighted average, given by:

(C1) $$Z_{eff}=\frac{\sum w_{i}Z_{i}}{\sum w_{i}},w_{i}=1/d$$

where $d_{i\;}$ is the distance of $Z_{i\;}$ to the assessment location for $Z_{eff\;}$. For the estimation of $Z_{i\;}$ the available GIS-datasets are used to define a closest matching Davenport classification (Wieringa, 1992). The resolution used for this assessment is 5 × 5 m^2^ that matches the resolution in which OpenStreetMap data is described for the modelling area. Using this resolution $Z_{eff\;}$ gives estimates to be used in Gaussian plume modelling with the footprint-based approach. However, this scale is not suitable for the Gaussian puff modelling, for which further averaging (up to 100 × 100m) is done. Technically, the assessment of $Z_{eff\;}$ is computationally costly, and therefore $Z_{eff\;}$ values for the modelling area have been computed and archived prior operational use for each wind direction in 10-degree intervals.

The assessment of $Z_{i\;}$ involves the GIS-datasets shown in Fig. 3, e.g., OpenStreetMap but requires complementary information as well, such as Sentinel 2 -based estimates for vegetation coverage. Details are not presented here; generally speaking, the approach yields URL values of 1–2 at urban areas and provides low values (0.0002) for sea areas and lakes. Due to the upwind directed approach however, low URL values are also obtained for roads when they are aligned parallel to the wind, which therefore impacts the modelling of, e.g., street canyons.

The third and final step of wind profiling involves the use of Eq. (3) to obtain a wind speed estimate for emission sources using their effective release height above ground. Further, the meteorology for each Gaussian puff being modelled is updated frequently, once every minute, or for every 100m travelled by the puff. Since the Gaussian dispersion equation is inaccurate for low wind speeds, a minimum wind speed of 1 m/s has been defined. As an additional simplification and to guarantee non-zero wind speeds at low heights above ground, a value of 0 is used for zero-plane displacement ($d_{0\;}$). The Gaussian dispersion formulas for the estimation of $c_{L}$ (Eq. (2)) do not address the influence of the change of the wind velocity with height between the emission source and the observation point.

### Limitations for the wind profiling approach

As noted in (Hammond et al., 2012) the estimation of $Z_{i\;}$ is challenging in real-world conditions. Even the definition for the surface area that is assessed in the aggregation is complicated; the use of a nested set of different elliptical upstream areas is suggested (Schmid and Oke, 1990). Therefore, the method presented here can be considered as a crude approximation. The performance of the presented method needs to be further developed and evaluated against wind measurements. Additionally, comparison against LES modelled wind fields (Hellsten et al., 2020) may provide useful information for further development.

## Footnotes

1 Traffic flow count data together with traffic congestion data (from www.here.com) are used for quality controlling traffic flow patterns the model uses.

2 A description in Finnish for the EMME model can be found from (Visited: February 1st, 2022): https://www.hsl.fi/sites/default/files/helsingin_seudun_tyossakayntialueen_liikenne-ennuste_kysyntamallit_5_2019.pdf.

3 Time estimate is based on data production rate for HMA, using 12-core 6/61 Broadwell 2400 MHz CPU with 32 GB of memory.
