# Supplementary material for: An operational urban air quality model ENFUSER, based on dispersion modelling and data assimilation
Source: Environ Model Softw. 2022 Oct;156:105460. doi: 10.1016/j.envsoft.2022.105460 (PMC9485198; doi:10.1016/j.envsoft.2022.105460)
Supplement: Multimedia component 1 [file mmc1.pdf]

SUPPLEMENTS - An operational urban air quality model ENFUSER, based on dispersion modelling and data assimilation

1. Footprint for Gaussian plume computations

In section 2, a Gaussian plume -shaped footprint was presented, aiming for a), the reduction of surface area needed to be assessed around each RP for Eq. 1, b), for allowing variable assessment resolution to exist within the assessed surface area and c), allow for efficient Eq. 2 computations. The presented footprint is formed by treating all surrounding surface area around RP as a collection of candidate areas in the size of 5 x 5 m^2. For each candidate their potential to impact concentration at RP is assessed. The footprint is the collection of candidate cells for which this potential exceeds a selected threshold.

To define the potential of a candidate cells for the footprint, unit emission sources with release height of 2m have been placed all around RP. Then given an arbitrary wind direction and speed, the contribution of each of the candidate cell's unit emitters has been assessed at RP; the footprint is formed to be the most impactful collection of cells that together constitute 99.9% of all concentration contributions at RP. In this assessment highly unstable stability class (A) and lowest allowed finite mixing height are used, which are the conditions requiring the largest and most detailed footprint. In other words, an omitted cell that is not included in the footprint can be safely excluded from the Eq. 1 assessment, as an emission source located at the cell's location cannot have a meaningful contribution to the concentrations at RP.

After the selection of cells to form the footprint, a merging operation is done to reduce the number of cells further. Afterwards, the cells that constitute the final footprint have variable area-of-effect depending on the cell's position; very close to the RP a higher resolution is used (no merging has been done), and the cell density is larger; farther away the area is covered by a smaller number of merged cells with larger area-of-effect. These cells with larger surface area are formed by merging the close by 5 x 5m^2 cells. The merging balances the relative potential of the footprint cells, but drastically reduces the number of cells. Assuming the area around RP is covered with unit emission sources, each cell regardless or its merged surface area have comparable concentration contributions at RP.

2. Gaussian plume precomputations

The whole footprint is aligned with the wind direction (upwind) in a single rotation operation. After this rotation, each cell has a specific location to check for emission sources (set K, which in turn yield the emission release rate Q for each $k \in K$). Each cell in the footprint can also assess Eq. 2 very efficiently, relying on pre-computed values, since:

1. For each cell the values for x_i, y_i (the location of the cell in the rotated coordinate system) are constant regardless of the rotation of the overall footprint.
2. The values of x_i, y_i can be readily used for Eq. 2 to assess the emission source's contribution at the RP due to geometrical symmetry.

3. The standard deviations (σ_y ,σ_z) are assessed with a constant value for x_i and they have a small number of possible states due to the use of a fixed set of stability classes.
4. In the modelling scope of ENFUSER the range of possible values of z and H for Eq. 2 are also limited.

The justification for the second property has been elaborated in Fig S1. As it can be seen from the figure, the location of the cell (x_i, y_i) can be directly used to compute the concentration caused by the emission source (ES) at the RP since the footprint has been rotated against the wind direction.

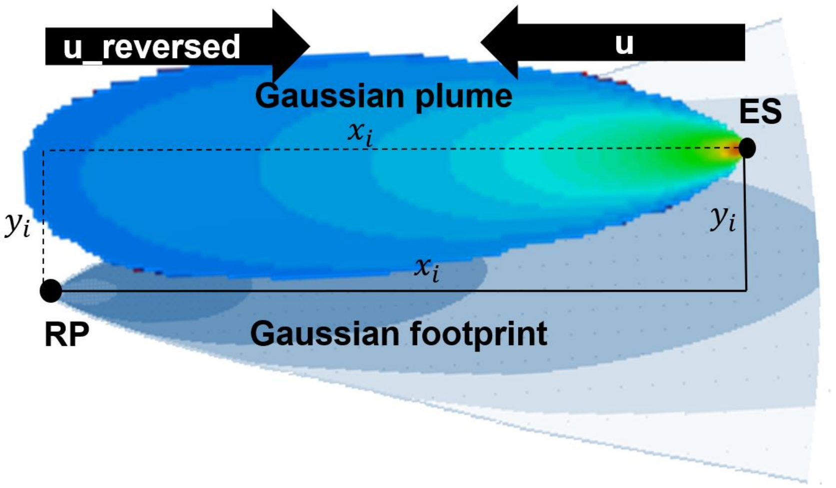

Figure S1: Illustration of the geometrical symmetry regarding footprint cell's location. Since the footprint is rotated to the upwind direction and the cell coordinates are unaffected by the rotation, the constant values of (x_i, y_i) yield the necessary spatial parameters for the estimation of Eq. 2.

Finally, for any cell all possible permutations for Eq. 2 without the term Q/u can be precomputed. The assessment of Eq. 2 then becomes an exercise of fetching a precomputed value as a function of z, H and stability class, and then multiplying this with Q/u.

2.1 Efficiency considerations for Gaussian puff

The aim of the puff dispersion modelling in ENFUSER is to address the dispersion of pollutants for which the footprint-based approach is less feasible or beyond reach. Generally, puff dispersion modelling can be regarded as an order of magnitude more costly computationally than Gaussian plume since a single emission source continuously releases new parcels of emissions to track and update. The computational burden is reduced by keeping the number of puffs low, and by streamlining the assessment of Eq. 1 or Eq. 4. The techniques adopted include:

- **Aggregation of emission sources.** Numerous incremental area emission sources (e.g., traffic, households, as 5 x 5m^2 area emission sources) are aggregated into much larger, e.g., 500 x 500m^2 bundles of emissions that are dealt as a single mixed emission source for puffs. The choice of resolution for emission source aggregation this way depends on the value of d_{cut}. Clearly, the chosen resolution must be significantly smaller than d_{cut}, for which a value of 2000m was used. A lower

number of emission source cells for Gaussian puff modelling means less new puffs to be released and modelled in every time step.

- **Puff merging.** Clearly diffused, closely packed groups of puffs can be merged. This can be used for e.g., shipping emissions where high release rate for puffs is initially useful for modelling accuracy; as the shipping emission puffs diffuse significantly, the gained accuracy is lost but the strain of having more puffs to assess remains. By merging such puffs together at a later stage this cost is avoided.
- **Puff removal.** Puffs that have clearly exit the modelling area can be completely omitted from the modelling in subsequent modelling time steps.
- **Reduction of RP's for Gaussian puff computations.** The Gaussian puff modelling layer has less spatial variability than the fine-scale Gaussian plume layer. This means that while the Gaussian plume layer is estimated with high resolution, a considerably coarser resolution can be applied with the Gaussian puff layer without any meaningful loss of accuracy.
- **Omission of puffs for set P.** For concentration computations with Eq.1 only the puffs near the RP are included to the set of P. Conversely, it is possible to exclude most of the puffs that do not have the potential to impact concentrations at the RP.
- **Use of precomputations.** The assessment of puff contributions to many different RP's can be organized in a way that facilitates the use of precomputations to a large extent, or that the bulk of the computations are done for each puff exactly once. This will improve the computational efficiency significantly as there can be hundreds of thousands of RP's for which Eq. 1 is used. Briefly put, for a given time each puff has a fixed travel distance that defines fixed values for gaussian standard deviations. The value of H, the puff's current centre point elevation, is constant. The value of z is also constant, i.e., the breathing height of 2m. The only RP-dependent computation involved is therefore the assessment of $exp\left(-d^2/4r_y\right)$ (Eq. A4).

3. Details for data assimilation

3.1 Persistent adjustments and the update procedure

The hourly adjustment set **a**, the outcome of an hourly data assimilation, is used to update a longer-term set of persistent adjustments (PA). The aim is to achieve long-lasting adjustments for emission sources that can also have an effect in forecasting when – naturally - no measurement evidence is present. The purpose of PA is also to eventually take over the main responsibility for emission factor adjustments; the significance of the hourly adjustments to model predictions should diminish once the PA stabilizes to a certain state that is in general agreement with the measurement evidence.

For each new hourly a_s obtained by the data assimilation procedure the corresponding value A_s is updated as follows:

$$A'_s = A_s[(1-v) + v\, a_s], s \neq BG \qquad (S1)$$

$$A'_{BG} = A_{BG}(1-v) + (a_{BG} + A_{BG})v$$

where A'_s is the updated value and v determines the update rate. A value of 0.01 is used for v. Initially, when no data assimilation has previously occurred for the pollutant species $A_{BG} = 0$ and $A_s = 1$ $(s \neq BG)$.

3.2 Diurnal variability for PA

Persistent adjustments must be prepared to differentiate the behaviour of emission sources for different times of day and day-of-week. For example, when the initial temporal pattern of a modelled emission source is incorrect, the update mechanism may correct the emission factors upwards for specific time of day, and downwards for another time of day. To address this possibility, the PA factors are also updated separately for different times-of-day and day-of-week. Technically, PA is managed separately for each pollutant species and emission source category, and for each of these there is a set of 24 x 3 diurnal values (working days, Saturdays, and Sundays, 24 values for each using local time). The time t is used to select one of the 72 persistent adjustment factors for the estimation of Eq. 5, and also for the selection of diurnal PA during update operation.

3.3 Continuity and setting the adjustment state range for background

For hourly regional background adjustment (a_{BG}), the assignment of possible set of discrete states prior the use of the data assimilation algorithm is different than it is for other source categories. For background a reasonable range of possible adjustments with e.g., 10 discretely defined values, is defined considering the historic background concentrations in the modelling area.

To enforce modelling continuity from a modelling hour to the next, the set of possible states are further limited based on the previous hour' data assimilation outcome, i.e., for any $a_s(t)$ the set of possible states is confined to be close to $a_s(t-1h)$. To enforce continuity further, the set M can include measurements from the previous hour and then assign a smaller weight w_m for these data points.

3.4 Omission of emission source categories due to inadequate information

Sometimes there can be emission source categories that do not provide meaningful contributions to model predictions in any measurement site's location. This can happen due to, e.g., low number of observation locations, less ideal wind direction with respect to the measurement sites, or simply due to low amount of emission releases for certain sources. In such cases WSSE of Eq. 6 can be insensitive for one or more a_s, which in turn reduces the reliability of the obtained value for a_s. Updating PA with unreliable data assimilation outcomes is not desirable. To address this issue, an additional rule to data assimilation algorithm has been defined; if an emission source contributes less than 5% of modelled concentrations across all the measurement locations on the average, then the source category will be excluded from the optimization task and will not therefore update PA.

3.5 Data assimilation for coarse particles

The modelling of PM_{10} involves the modelling of fine particles and coarse particles ($PM_{coarse} := PM_{10} - PM_{2.5}$) separately, for which different dispersion formulas are used (Appendix A). However, measurement data or

regional background for PM_{coarse} often do not exist explicitly, and these need to be proxied. This is done simply by subtracting $PM_{2.5}$ from PM_{10} in AQ measurement locations where both pollutant types are being measured. For the regional background the approach is the same, but the subtraction is performed across the whole modelling area to obtain a proxy for PM_{coarse}. After dispersion modelling and data assimilation, adjusted PM_{10} model predictions are then obtained by summing the adjusted model predictions of $PM_{2.5}$ and PM_{coarse}.

4. Statistical parameters used for evaluation

The statistical formulas used in the evaluation of model performance have been introduced in this section. First, we define a couple of useful variables for the statistical formulas as follows:

P_i = Predicted hourly concentration [µgm-3]

$\bar{P}$ = Mean predicted hourly concentration over N data points [µgm-3]

O_i = Observed hourly concentration [µgm-3]

$\bar{O}$ = Mean observed hourly concentration over N data points [µgm-3]

For simplicity the sum operation $\sum_{i=1}^{N}$ is expressed as $\sum$ in the formulas below.

Fractional Bias (FB) varies between -2 and 2. For a model the ideal value is zero, which occurs when the average prediction equals the average observation. A strong negative value indicates that the model is systematically overpredicting, and strong positive value indicates systematic underpredictions respectively. FB is given by:

$$2\frac{(\bar{O}-\bar{P})}{(\bar{O}+\bar{P})}$$

Factor-of-two (F2) varies between 0 and 1. A value of 1 indicates that the model predictions are always lower or equal to double the observed value, and at least half of the observation value. F2 is defined as follows:

$$\frac{1}{N}\sum f(P_i, O_i)$$

where $f(P_i, O_i) = 1$ when $0.5 \le \frac{P_i}{O_i} \le 2$, otherwise $f(P_i, O_i) = 0$

Normalized Mean Squared Error (NMSE) measures the variability of model predictions against observations. It is like the statistical measure of Mean Squared Error (MSE), however NMSE is in proportion to the average observed and modelled values, hence facilitating NMSE to be a standardized measure for model performance. A low value of NMSE is considered ideal for a model. NMSE is given by:

$$\frac{\overline{(O_i-P_i)^2}}{\bar{O}\bar{P}} = \frac{\frac{1}{N}\sum(O_i-P_i)^2}{\bar{O}\bar{P}}$$

Pearson Correlation Coefficient (PCC) is a correlation coefficient used in linear regression. The formula returns a value between -1 and 1, where 1 indicates a strong positive relationship, -1 indicates a strong negative relationship. A value of zero indicates no relationship at all. PCC is given by:

$$\frac{\sum(O_i - \bar{O})(P_i - \bar{P})}{\sqrt{\sum(O_i - \bar{O})^2 \sum(P_i - \bar{P})^2}}$$

Index of Agreement (IA) is a standardized measure of the degree of model prediction error which varies between 0 and 1. The agreement value of 1 indicates a perfect match, and 0 indicates no agreement at all. IA is given by:

$$1 - \frac{\sum(P_i - O_i)^2}{\sum(|P_i - \bar{O}| + |O_i - \bar{O}|)^2}$$
